# New Hybrid Combination for Local Crucian Carp Germplasm Improvement: Dongting Lake Crucian Carp (♀) × Hefang Crucian Carp (♂)

**DOI:** 10.3390/ijms27115049

**Published:** 2026-06-03

**Authors:** Liming Shao, Haiqi Li, Haipeng Guo, Yi Zhou, Kaikun Luo, Wuhui Li, Chongqing Wang, Jing Wang, Liang Guo, Qingfeng Liu, Qinbo Qin, Zhongyuan Shen, Shaojun Liu

**Affiliations:** 1 Engineering Research Center of Polyploid Fish Reproduction and Breeding of the State Education Ministry, College of Life Sciences, Hunan Normal University, Changsha 410081, China; slm@hunnu.edu.cn (L.S.); 202420142924@hunnu.edu.cn (H.L.); 202470142963@hunnu.edu.cn (H.G.); 18175526196@163.com (Y.Z.); 18008403844@163.com (K.L.); liwuhui11@163.com (W.L.); wcq@hunnu.edu.cn (C.W.); hwangjing0826@163.com (J.W.); hnsfgf@hunnu.edu.cn (L.G.); liuqingfeng@hunnu.edu.cn (Q.L.); qqb@hunnu.edu.cn (Q.Q.); 2 Yuelushan Laboratory, Changsha 410128, China; 3 State Key Laboratory of Developmental Biology of Freshwater Fish, Hunan Normal University, Changsha 410081, China

**Keywords:** distant hybridization, fish germplasm improvement, 5S rDNA, mtDNA

## Abstract

Due to overfishing, eutrophication of rivers and lakes, and irrational stocking practices, the diversity of wild native carp populations has declined, leading to germplasm degradation and a decrease in fish quality, thereby affecting the sustainable development of fisheries. In this study, a novel hybrid crucian carp lineage (designated LWR) was successfully established via distant hybridization using Dongting Lake crucian carp (LC, ♀) and Hefang crucian carp (WR, ♂) as parental stocks. Systematic analyses were conducted on the morphology, ploidy, fertility, growth performance, survival rate, and molecular genetics of LWR. The results reveal that LWR is an allodiploid (2n = 100), with a chromosome number identical to that of its parents. Gonadal development in the hybrid progeny (LWR) was normal, with both sexes being fertile and reaching sexual maturity at one year of age. Morphologically, LWR exhibited intermediate traits with a paternal bias, characterized by a deep-bodied and elongated shape. In terms of growth performance, LWR displayed significant heterosis (approximately 145% and 271% higher than the body weight of the maternal parent at 6 months and 1 year). Molecular genetic analysis indicated that the 5S rDNA sequences of LWR were predominantly inherited from the paternal parent WR, with insertions, deletions, recombination, and mutations detected. The mtDNA sequences exhibited 99.78% similarity to those of the maternal parent LC, following maternal inheritance with sporadic nucleotide variations. These findings offer a new paradigm for hybridization and a theoretical foundation for the improvement and sustainable utilization of indigenous crucian carp germplasm resources, the selective breeding of improved aquaculture strains, and the sustainable development of fisheries.

## 1. Introduction

In aquaculture, hybridization has become an important strategy for breeding and germplasm improvement. Distant hybridization, in particular, enables the transfer of the genome from one species to another, thereby inducing alterations in genotype and phenotype. Offspring derived from distant hybridization often exhibit heterosis in growth rate, survival rate, and disease resistance [1,2]. For instance, distant hybridization between Japanese white crucian carp (*Carassius cuvieri*) and red crucian carp (*Carassius auratus* red var.) has produced the Hefang crucian carp strain, which possesses superior traits [2]. In this strain, mutant genes and chimeric genes closely associated with these advantageous characteristics have been identified [3,4,5]. Smith et al. [6] conducted interspecific hybridization between white bass (*Morone chrysops*) and striped bass (*Morone saxatilis*), producing hybrids that exhibited rapid growth and tolerance to extensive culture conditions, with clear heterosis. Similarly, Xiao et al. [7,8] hybridized blunt snout bream (*Megalobrama amblycephala*) and topmouth culter (*Culter alburnus*) to obtain a novel hybrid fish characterized by rapid growth, high flesh quality, and strong stress tolerance. Subsequently, they established a bisexual fertile allodiploid hybrid lineage derived from this hybridization [9].

Dongting Lake native crucian carp (*Carassius auratus*, LC) (wild crucian carp from Dongting Lake) belongs to the *Family Cyprinidae* and the *Genus Carassius*. It has a grayish-brown body coloration and an elongated body shape. Adult fish typically measure 10–19 cm in body length and weigh 75–150 g. This species is omnivorous, highly adaptable, and reaches sexual maturity within one year, although its growth rate is slow [10]. Previous studies have shown that native crucian carp exhibit multiple ploidy populations: diploid (2n = 100) individuals constitute the main population, while triploid (3n = 150) and a small number of tetraploid (4n = 200) individuals also exist, with no obvious morphological differences among the three ploidy types [10]. However, previous research on Dongting Lake native crucian carp has primarily focused on aspects such as intestinal microbial diversity [11] and molecular biological characteristics [10], with relatively few studies addressing germplasm improvement. According to regional fishery resource surveys and published reports [10,12,13,14], overfishing, eutrophication of rivers and lakes, and irrational stocking practices have contributed to a decline in the diversity of wild native crucian carp populations in the Dongting Lake water system and adjacent basins. These changes have been associated with reduced genetic diversity (e.g., loss of microsatellite alleles and decreased proportion of diploid individuals) [10,12], lower average body condition, and decreased commercial catch per unit effort [13,14]. Consequently, germplasm degradation and a decrease in fish quality have been observed, which may affect the sustainable development of fisheries [15,16,17]. There is an urgent need to protect the germplasm resources of native crucian carp through conservation measures such as habitat restoration and reducing fishing pressure on wild populations. Developing superior aquaculture strains plays a significant role in alleviating the fishing pressure on wild populations.

Hefang crucian carp (2n = 100), developed by Professor Shaojun Liu’s team at Hunan Normal University, exhibits a bluish-gray body color and a deep-bodied shape. At one year of age, adult fish reach a body length of 16–23 cm and an average weight of 350 g. This strain is characterized by rapid growth, strong stress tolerance, abundant flavor amino acids, and delicious flesh quality [18,19], all of which have been consistently observed under standardized culture conditions (20–28 °C, dissolved oxygen > 5 mg/L, pH 7.0–8.5, with formulated feed). Quantitatively, WR shows 20–30% higher growth, significantly elevated flavor amino acid levels, and better survival than common crucian carp [18,19]; its rapid growth is partly due to low *mstnb* expression [20], making it a high-quality crucian carp germplasm. In the present study, distant hybridization was carried out using Dongting Lake native crucian carp as the maternal parent and Hefang crucian carp as the paternal parent, with the aim of improving the germplasm of Dongting Lake native crucian carp. To investigate the genetic and molecular biological characteristics of the novel hybrid, we analyzed the ploidy, fertilization rate, hatching rate, survival rate, fertility, growth rate, morphological traits, 5S rDNA, and mtDNA of the hybrid progeny. This research provides a new hybrid germplasm (LWR) for the aquaculture industry, which represents an effective approach for improving the growth and survival traits of local crucian carp germplasm, and proposes a novel hybrid combination through germplasm improvement, thereby supporting the sustainable development of fisheries and enhancing the economic efficiency of aquaculture.

## 2. Results

### 2.1. Formation and Morphological Characteristics of the Hybrid Progeny (LWR)

**Figure 1:**
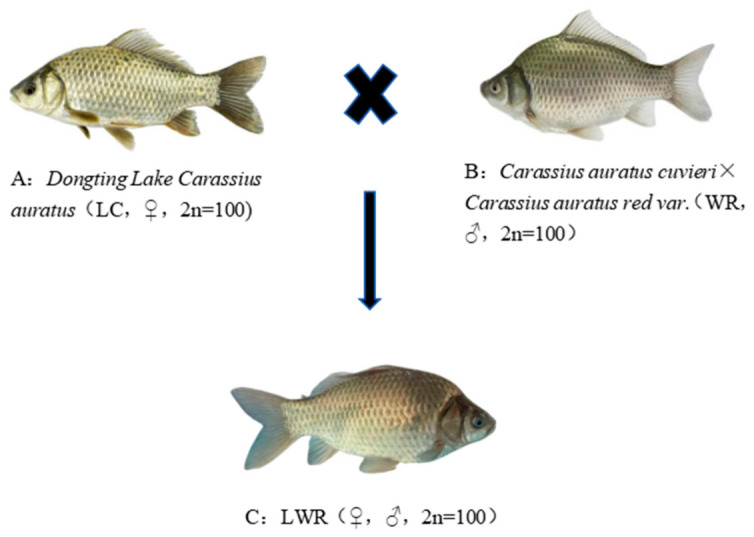
Breeding diagram of hybrid F_1_ generation ((**A**): LC, (**B**): WR, (**C**): LWR).

The hybridization process between Dongting Lake native crucian carp (LC) and Hefang crucian carp (WR), as well as the formation of the hybrid progeny (LWR), is illustrated in Figure 1. Preliminary observations on the external morphology of LC, WR, and LWR revealed that all three lacked barbels. LC exhibited a laterally compressed and relatively elongated body, with a flat and narrow abdomen and a gently arched back; its body color was brownish. WR had a rounded abdomen, a shorter body length than LC, a prominently arched back characterized by greater body depth, and a bluish-gray body color. LWR displayed a laterally compressed body with a rounded abdomen, an intermediate body length between the two parents, and a distinctly arched back, inheriting the greater body depth characteristic of WR. The body color of LWR was brownish, showing similarities to both LC and WR, indicating that while LWR inherited morphological traits from both parents, certain variations also emerged.

**Figure 2:**
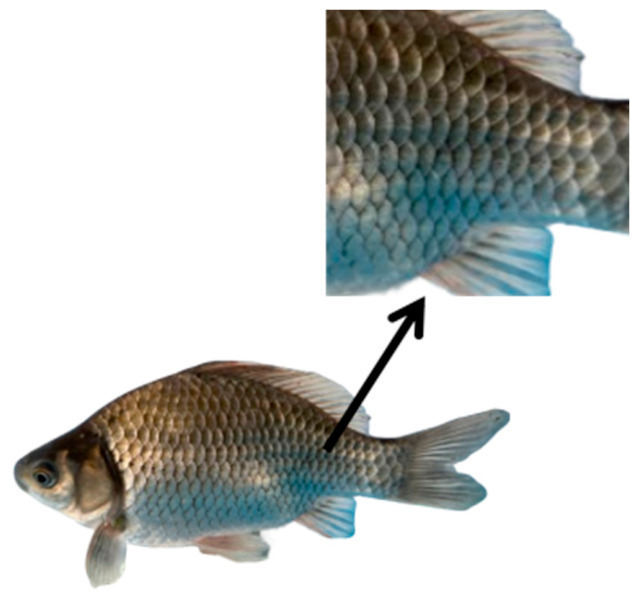
Diagram of deformities in hybrid F_1_ generation fish.

The fertilization and hatching rates of LWR were 89.3% and 78.7%, respectively (For comparison, historical data from our laboratory, obtained under similar but not identical conditions, showed fertilization rates of 84% for LC and 90.2% for WR, and hatching rates of 71% for LC and 81.5% for WR. These historical values are provided for reference only, as they were not generated from parallel experiments). Among LWR juveniles, some malformed individuals were observed, with a malformation rate of 1.2%; during subsequent rearing, the mortality rate of these malformed individuals reached 91.8%. Adult malformed LWR individuals are shown in Figure 2, characterized by lateral curvature and folding of the caudal vertebrae, with most exhibiting a spiral swimming pattern.

The meristic traits of LC, WR, and LWR are presented in Table 1, and the morphometric traits in Table 2. The number of lateral line scales in LWR (28–31) was generally consistent with that in LC (27–30) but lower than that in WR (30–31). The body length/body height ratio of LC (3.41 ± 0.10) was significantly higher than that of WR (2.21 ± 0.13) and LWR (2.35 ± 0.07) (*p* < 0.05). The body length/head length ratio of LWR (3.54 ± 0.13) was significantly lower than that of LC (3.83 ± 0.12) and WR (3.73 ± 0.16). The head length/head height ratio of LWR (1.22 ± 0.07) was significantly higher than that of LC (1.10 ± 0.02). Collectively, LWR exhibited morphological characteristics more similar to WR while also displaying distinctive hybrid traits.

### 2.2. Ploidy and Fertility of LWR

**Figure 3:**
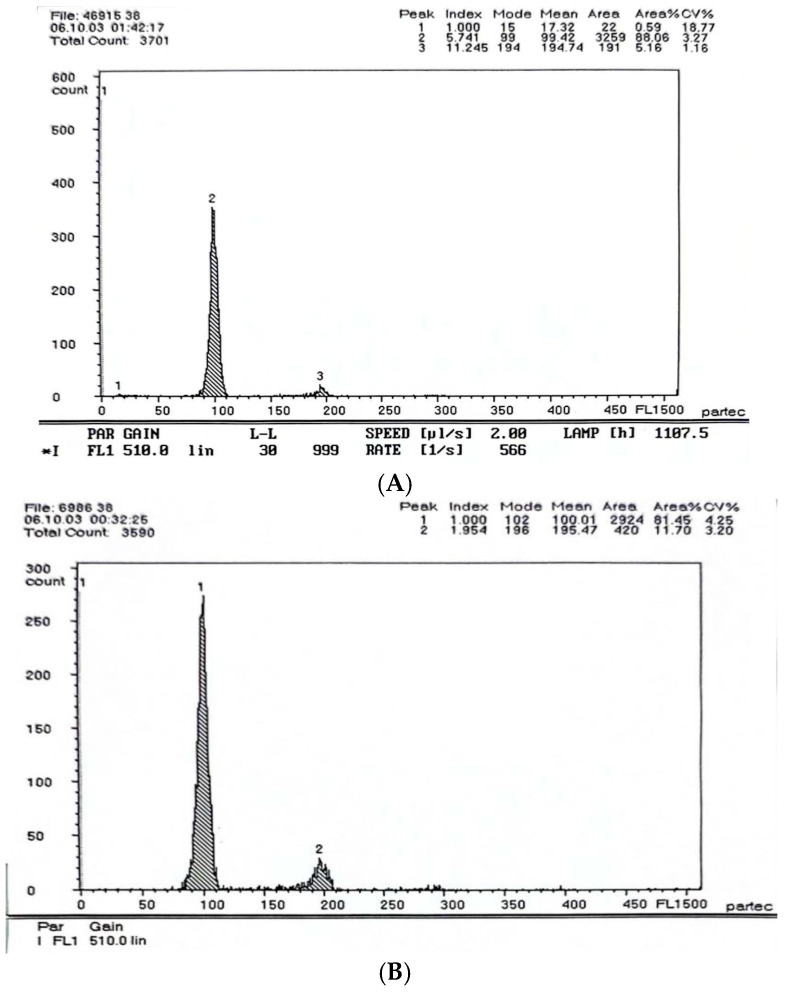

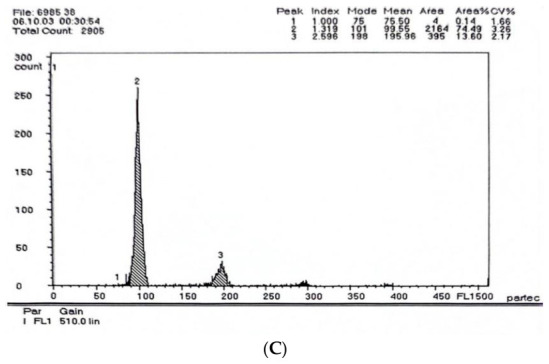
Cytometric histograms of DNA fluorescence of LC, WR and LWR ((**A**): LC; (**B**): WR; (**C**): LWR).

**Figure 4:**
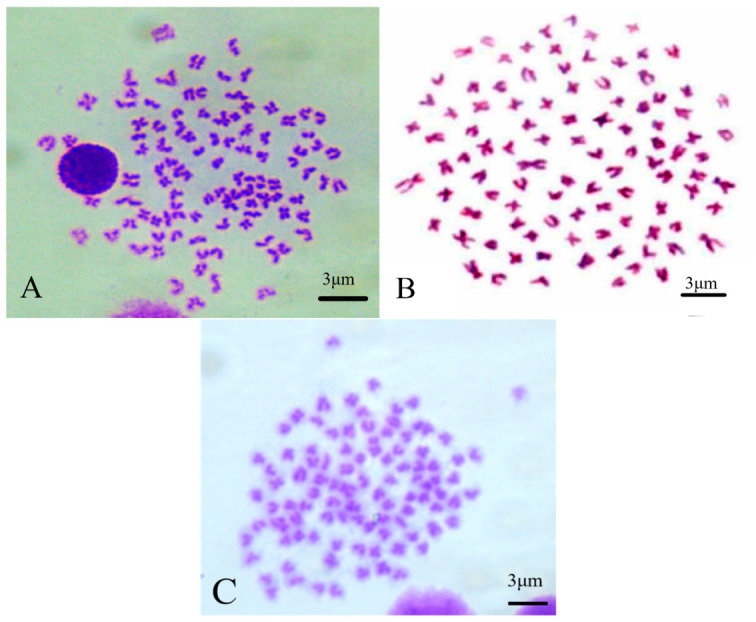
Chromosome distribution of LC, WR and LWR ((**A**): LC; (**B**): WR; (**C**): LWR, Bar = 3 μm).

Using RCC (red crucian carp, *Carassius auratus* red var., 2n = 100) as a reference, we compared the DNA content of LWR with that of LC and WR (Figure 3, Table 3). A sample was classified as diploid if the ratio of its mean DNA content to that of RCC fell within 0.95–1.05. The mean DNA content ratios for LC, WR, and LWR were 0.996, 1.001, and 0.997, respectively, all within this threshold, indicating that LWR is an allodiploid fish. Chromosomal metaphase spreads of LC, WR, and LWR were obtained by preparing chromosome slides from kidney cells (Figure 4). The results show that LC, WR, and LWR each possessed 100 chromosomes, all being diploid, consistent with the DNA content measurements.

**Figure 5:**
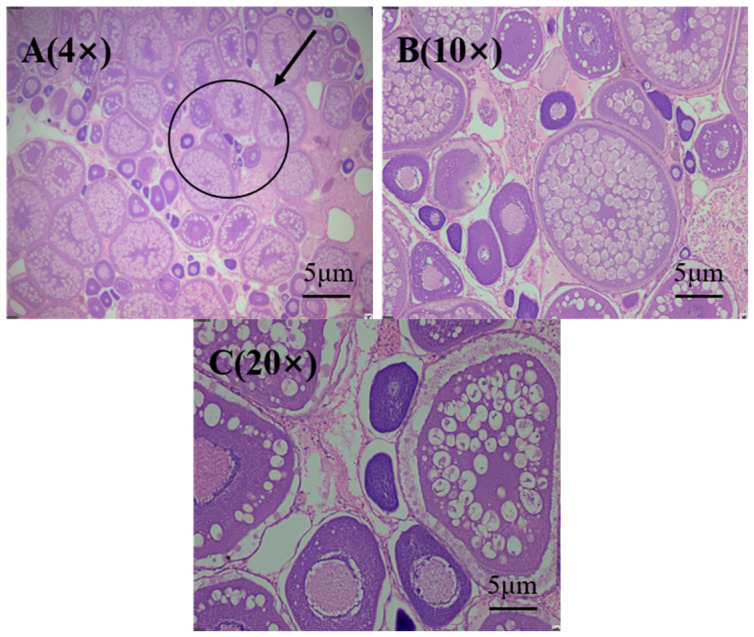
Gonad histology section image of LWR for 1 year ((**A**–**C**): The ovarian microstructure of LWR; Bar = 5 μm). The circles and arrows indicate stage II, stage III, and stage IV oocytes.

**Figure 6:**
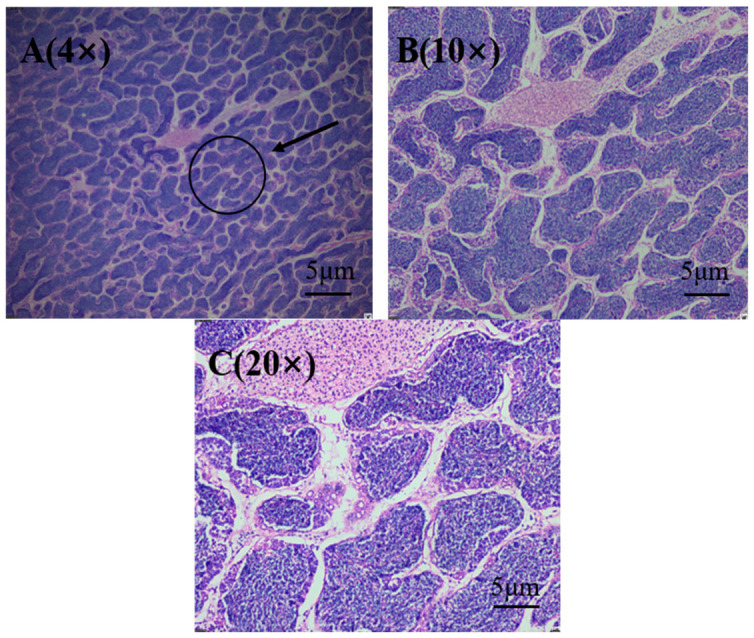
Gonad histology section image of LWR for 1 year (**A**–**C**): The sperm microstructure of LWR; Bar = 5 μm. The circles and arrows indicate the testicular lobules, which contain abundant normally developed and mature spermatozoa.

Regarding fertility, paraffin section results of gonadal tissues from one-year-old LWR are shown in Figure 5 and Figure 6. In the ovaries, stage IV oocytes were observed, characterized by irregularly oval nuclei with folded nuclear membranes, a thickened zona radiata, and gradual filling of yolk granules. Stage II and stage III oocytes were also present, with nuclei exhibiting weak basophilia, a thinner zona radiata, a follicular layer composed of two layers of flattened cells, and initial yolk deposition in the peripheral ooplasm, indicating normal and fully mature ovarian development. In the testes, numerous testicular lobules containing normally developing and mature spermatozoa were observed, indicating normal testicular development. During the breeding season, artificial dry insemination was performed on F_1_ LWR individuals, and both females and males could successfully release eggs and milt. Together, these results demonstrate that LWR is a bisexually fertile lineage that reaches sexual maturity at one year of age, consistent with the parental species.

### 2.3. Structural Analysis of 5S rDNA Sequences in LWR

**Figure 7:**
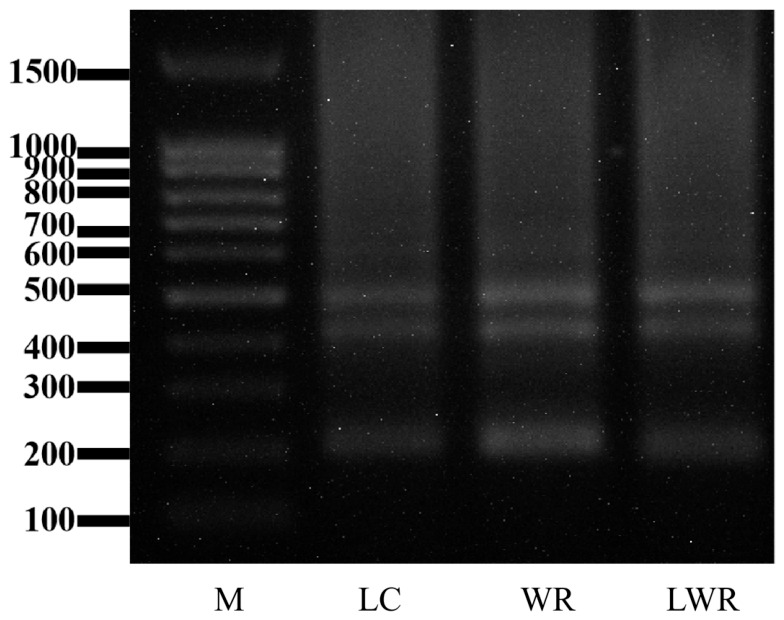
Comparison of 5S amplification products.

**Figure 8:**
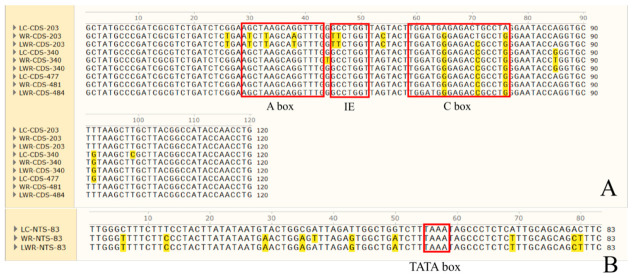

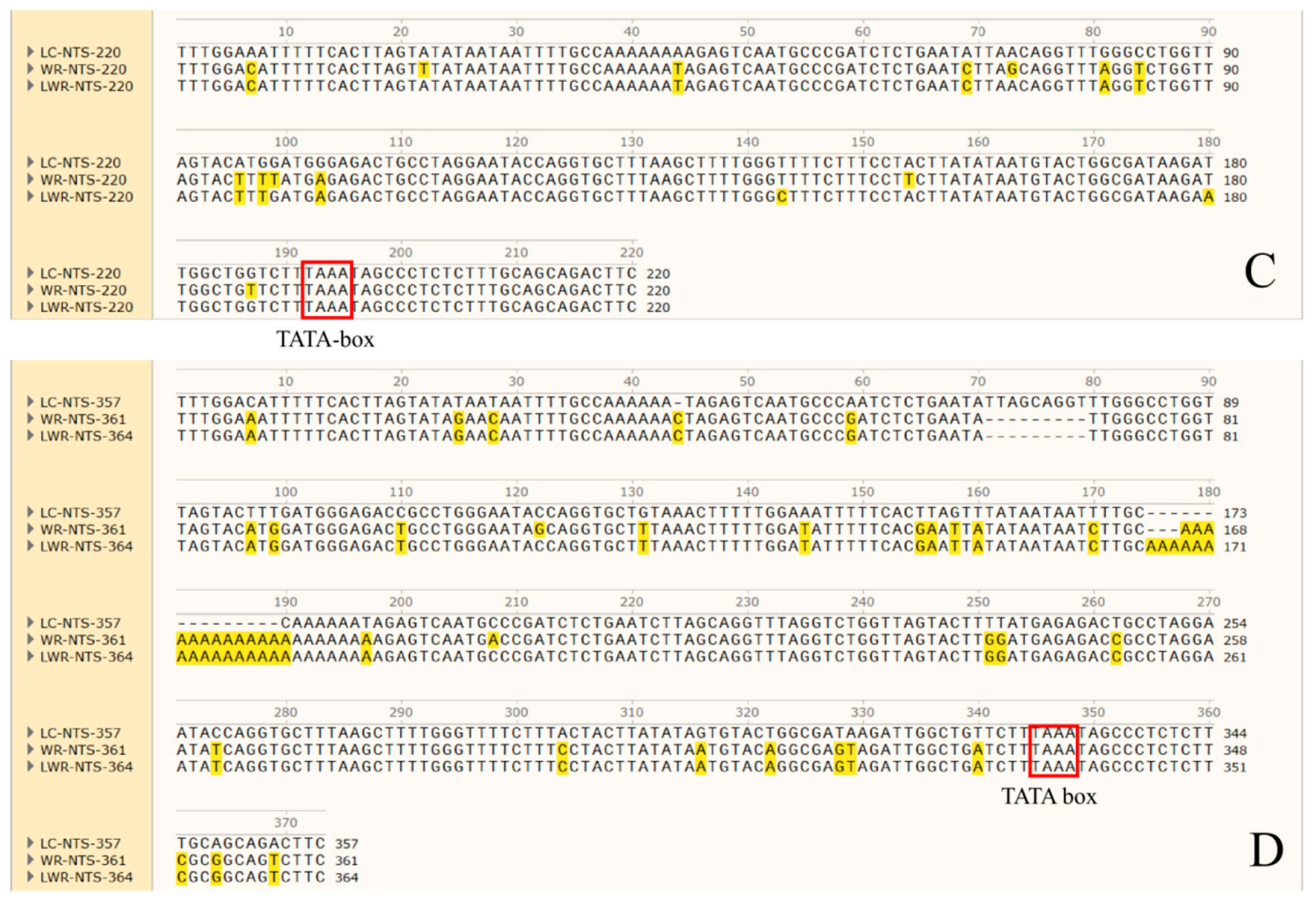
Comparative analysis of 5S rDNA from LC, WR and LWR. (**A**) Comparison of the 5S rDNA coding regions from LC, WR, and LWR. ICRs are included in the boxes. (**B**–**D**) Comparison of the NTS sequences from LC, WR, and LWR. The NTS upstream TATA-like sequences are included in the boxes.

Using 5S rDNA primers, we amplified and sequenced LC, WR, and LWR. The results show that LC exhibited three bands (203, 340, and 477 bp), WR exhibited three bands (203, 340, and 481 bp), and LWR exhibited three bands (203, 340, and 484 bp) (Figure 7). BLASTn analysis indicated that all bands corresponded to 5S rDNA repeat units, each consisting of a 3′ coding region, a nontranscribed spacer (NTS), and a 5′ coding region. The structures and homologies of the 5S rDNA sequences of LC, WR, and LWR are shown in Figure 8 and Figure S1. The results revealed that the 203 bp fragment in LWR was inherited from the paternal WR 203 bp fragment, with 98.52% similarity between the two. Similarly, the 340 bp and 484 bp fragments were inherited from the corresponding WR 340 bp and 481 bp fragments, with similarities of 97.35% and 98.97%, respectively, indicating a bias toward paternal inheritance. The 5S rDNA fragments of LWR (designated 203, 340, and 484 bp) corresponded to distinct NTS types: NTS-83, NTS-220, and NTS-364, representing fragments of 83, 220, and 364 bp, respectively. The A box, IE, and C box were identified within the coding regions (Figure 8A). A TATA box was identified in all NTS sequences and was modified to TAAA (Figure 8B–D). Collectively, the coding regions and NTS sequences of LWR inherited genetic characteristics from both parents, accompanied by certain mutations and recombination events.

### 2.4. Mitochondrial DNA Structure of LWR

**Figure 9:**
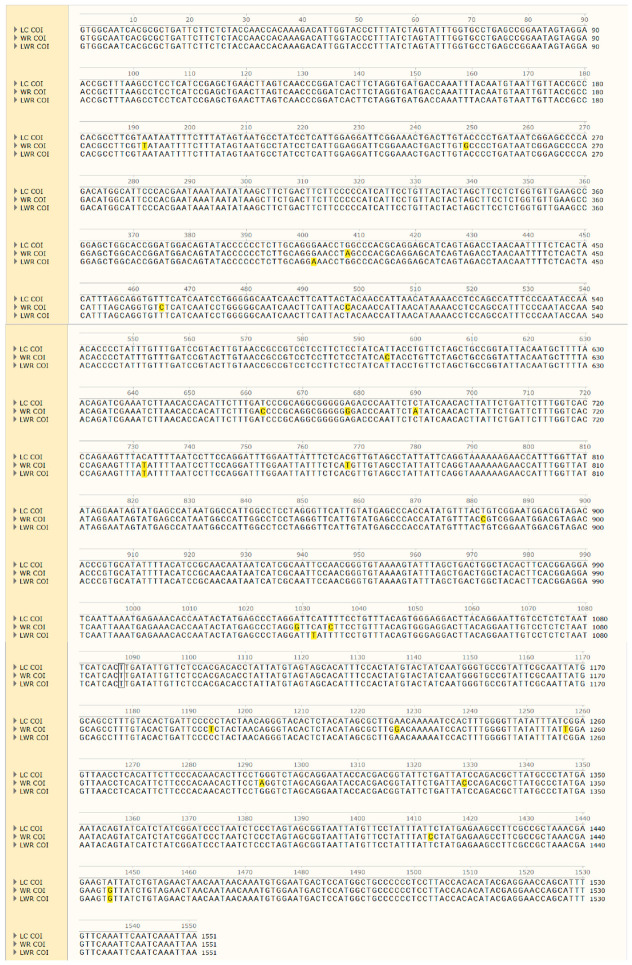
*COI* gene sequences for LC, WR and LWR.

**Figure 10:**
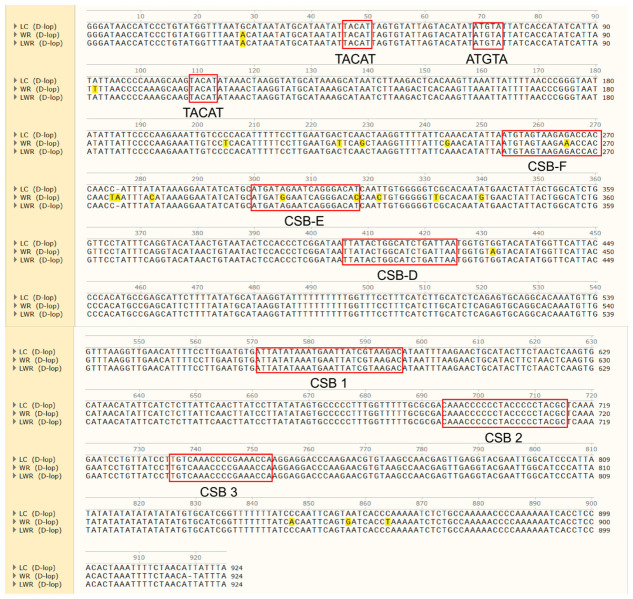
Sequence alignment of the D-loop regions of LC, WR, and LWR. The positions of conserved sequence blocks (CSB-D, CSB-E, CSB-F, CSB-1, CSB-2, CSB-3) and the core sequence (TACAT) are boxed. All CSB motifs in LWR are identical to those of LC.

Mitochondrial sequence analysis revealed that the complete mtDNA of LWR is 16,580 bp in length and comprises two rRNA genes, 13 protein-coding genes, 22 tRNA genes, and non-coding regions (the D-loop and the light-strand replication origin) (Figure S2). The structural features of the LWR mtDNA are summarized in Table 4. Analysis of the complete LWR mtDNA sequence showed an A + T content of 57.74% and a G + C content of 42.26%, indicating a pronounced AT bias. The mtDNA sequence similarity between LWR and LC was 99.78%, whereas that between LWR and WR was 98.85%, demonstrating that the mtDNA of LWR strictly follows maternal inheritance. The *COI* and D-loop regions in the LWR mitochondrial genome exhibited inheritance of genetic characteristics from the maternal species, along with certain nucleotide mutations or insertions/deletions. The *COI* gene sequences of LC, WR, and LWR are presented in Figure 9. Within the D-loop region, the core sequence (TACAT), its reverse complement (ATGTA), and conserved sequences (including CSB-D, CSB-E, CSB-F, CSB 1, CSB 2, and CSB 3) were identified (Figure 10). All six CSB motifs remained intact, with no deletions or rearrangements. Sequence alignment revealed that these motifs in LWR were identical to those of the maternal parent LC. The few nucleotide substitutions (one transition) observed between LWR and LC were all located outside the conserved CSB regions and therefore did not affect the structural integrity of these motifs.

### 2.5. Growth Rate and Survival Rate of LWR

Under identical rearing conditions, body weight was measured for LC, WR, and LWR at six months and one year of age, with the results presented in Table 5. The mean body weight of LWR (43.4 ± 5.5 g at six months; 297.0 ± 9.2 g at one year) was significantly higher than that of the maternal parent LC (17.7 ± 2.9 g at six months; 79.95 ± 8.2 g at one year) and slightly lower than that of the paternal parent WR (51.1 ± 1.1 g at six months; 335.5 ± 0.7 g at one year). At six months of age, LWR exhibited a body weight 145.2% greater than that of LC and 15.1% less than that of WR; at one year of age, LWR showed a body weight 271.5% greater than that of LC and 11.5% less than that of WR, reflecting its hybrid nature. These results indicate that LWR has inherited the growth advantage of the paternal parent WR.

Additionally, the survival rates of the hybrid F_1_ generation (LWR) were recorded at 15 days, 1 month, 3 months, 6 months, and 1 year of age, and the results are presented in Table 6. The survival rate at 15 days was 91.9%, which decreased to 84.6% by 1 month of age, representing a decline of 7.3 percentage points. At 3 months, 6 months, and 1 year of age, the survival rates were 81.2%, 80.2%, and 79.7%, respectively, with the decreases narrowing to 3.4, 1.0, and 0.5 percentage points, respectively. At 1 year of age, the survival rate of the hybrid F_1_ (LWR) remained at a relatively high level of 79.7%, indicating that the hybrid F_1_ (LWR) possesses excellent overall survival performance, good long-term survival potential, and higher survival rate, thereby demonstrating heterosis. Collectively, the survival curve of LWR approximates a “convex” type, in which mortality is mainly concentrated in the early life stages, while the survival rate remains relatively stable after reaching adulthood.

## 3. Discussion

Distant hybridization is an important technique for preventing germplasm degradation and facilitating varietal improvement [21,22,23,24]. Previous studies have demonstrated that a series of high-quality improved fish strains have been generated through distant hybridization. For instance, our laboratory has developed new varieties via distant hybridization, including the Hefang crucian carp series [25,26,27], disease-resistant grass carp [28], and the hybrid (*blunt snout bream* ♀ × *topmouth culter* ♂) × male blunt snout bream [29]; all of these exhibit significant advantages over their parents in growth rate, disease resistance, and flesh quality. Additionally, improved local strains with enhanced stress tolerance have been produced, such as the red mirror carp (*Xingguo red carp* ♀ × *Soviet mirror carp* ♂), Yue carp (*pouch red carp* ♀ × *Xiangjiang wild carp* ♂), and cold-tolerant pouch red carp (*Heilongjiang wild carp* ♀ × *pouch red carp* ♂) [30]. Extensive and systematic research on distant hybridization in fish has provided valuable insights into the genetic characteristics of hybrid offspring [21,31,32,33,34]. In the genetic model of distant hybridization, crosses between different strains often yield offspring exhibiting heterosis [1,2]. In the present study, we obtained an allodiploid hybrid progeny (LWR, 2n = 100) through distant hybridization between Dongting Lake native crucian carp (LC, ♀) and Hefang crucian carp (WR, ♂). At six months of age, the average body weight and survival rate of LWR were 43.4 ± 5.5 g and 80.2%, respectively; at one year of age, these values were 297.0 ± 9.2 g and 79.7%, respectively. These results indicate that the hybrid progeny clearly inherited the rapid growth advantage of the paternal parent while exhibiting higher survival rates under natural outdoor conditions, suggesting potentially improved stress resistance compared with the maternal parent LC. However, direct stress challenge tests (e.g., temperature, hypoxia, or pathogen challenge) are needed to confirm this observation.

Furthermore, distant hybridization is often associated with phenotypic alterations [35,36,37,38]. Our study revealed that the number of dorsal fin rays in LWR was intermediate between that of LC and WR, while other meristic traits showed similarity to either the paternal or maternal parent (Table 1). Notably, LWR exhibited the deep-bodied characteristic of WR, with a significant difference in the body length/body height ratio between LWR and LC (*p* < 0.05). Meanwhile, LWR also possessed traits not found in either parent: the body length/head length ratio of LWR differed significantly from both parents (*p* < 0.05), and a significant difference was also observed in the head length/head height ratio between LWR and LC (*p* < 0.05). These findings suggest that the paternal parent WR played a substantial role in shaping the traits of the hybrid progeny LWR (Table 2), which displayed pronounced hybrid characteristics. Interestingly, under identical rearing conditions, the body color of LWR was intermediate between that of LC and WR, appearing brownish—darker than the brown of LC but lighter than the bluish-gray of WR. In terms of body shape and color, LWR more closely resembled WR. (Limitations regarding color assessment: The body color comparison in this study was based on visual observation without the use of a standardized color chart or colorimeter. Although three independent observers reached a consensus, the description is inherently subjective. Future studies should employ objective methods such as a colorimeter or digital image analysis to quantify body color in hybrid offspring.) Collectively, LWR exhibited distinct hybrid characteristics in its external appearance. Notably, a certain proportion of malformed individuals were observed among the hybrid progeny, which we hypothesize may be attributable to biological differences between the two parents, genomic divergence, and the genetic distance between them—factors that also contribute to the outstanding traits of hybrid offspring [35,39,40,41].

At six and twelve months of age, the average body weight of LWR was significantly higher than that of LC (*p* < 0.05) but slightly lower than that of WR (Table 4). Previous studies have shown that low expression of the *mstnb* gene contributes to rapid growth in WR [20]. Although we did not directly examine *mstnb* expression in LWR, it is possible that the rapid growth of LWR is also associated with *mstnb* regulation inherited from the paternal parent. This hypothesis, however, requires direct experimental validation (e.g., qPCR, Western blot) in future studies [20,42,43,44,45].

The combination of parental genomes in distant hybridization can lead to alterations in gene structure and expression in hybrid offspring [46,47,48,49]. Findings from hybridization experiments based on genomic or transcriptomic studies have revealed the presence of mutated and chimeric genes in hybrids [4,50]. In the present study, recombination and mutations were also observed in the 5S rDNA of the hybrid progeny LWR (Figure 8A–D). Specifically, nucleotide positions 38 and 71 in the CDS-203 sequence, as well as positions 143 and 180 in the NTS-220 sequence, exhibited mutations distinct from both parents. In the NTS-364 sequence, a deletion of a nucleotide segment (TTAGCAGGT) was observed at positions 70–78 compared with the maternal parent, consistent with the paternal parent, while an insertion of an identical segment (AAAAAAAAAAAAA) occurred at positions 169–181, also matching the paternal parent. Unlike the paternal parent, however, an additional three-nucleotide insertion (AAA) was present at positions 166–168. Notably, these changes did not represent complete inheritance of the parental genomes but were characterized by heritability, variability, and heterozygosity, consistent with findings reported by Liu et al. [51]. As important markers of species variation and evolution in fish, 5S rDNA sequences often undergo nucleotide recombination in many hybrid progeny [52,53].

Fish mitochondrial DNA (mtDNA) serves as an effective tool for studying fish genetics and population genetics, as mtDNA is highly conserved across different fish species and often maintains sequence consistency [54,55,56,57]. Our study demonstrated that the mtDNA sequence of LWR was most similar to that of LC, consistent with the maternal inheritance pattern of mtDNA and aligning with previous findings [35,58,59]. Additionally, compared with the parental mtDNA sequences, LWR exhibited certain nucleotide mutations and insertions or deletions. We hypothesize that this may be related to the disruption of the long-term co-evolutionary relationship between the nuclear and mitochondrial genomes during distant hybridization [60,61,62,63]. However, the mtDNA sequence of LWR was 99.78% identical to that of LC and only 98.85% identical to that of WR, clearly supporting strict maternal inheritance. The few nucleotide substitutions between LWR and LC are likely spontaneous mutations or nuclear-mitochondrial incompatibilities. No direct evidence of paternal mtDNA leakage was obtained in this study. Numerous studies have demonstrated that the *COI* gene sequence can be used as a DNA barcode for fish species identification and phylogenetic analysis at the species level [64,65], while the D-loop region (also known as the control region) evolves rapidly and exhibits high sequence polymorphism, making it the most sensitive genetic barcode for distinguishing individuals or closely related species within mtDNA [66,67,68,69]. Therefore, both *COI* and the D-loop region can be used to analyze the degree of similarity or differentiation between LWR and its parents. Our results show that both 5S rDNA and mtDNA in LWR exhibited differences from the parents, including various nucleotide mutations and recombinations, which can serve as reliable molecular markers for LWR identification.

Survival rate statistics revealed that the F_1_ hybrids in this study exhibited significantly higher survival rates (91.9%, 84.6%, 81.2%, 80.2%, and 79.7%) during both the juvenile and adult stages compared with several previously reported hybrid combinations. In the present study, LWR exhibited a one-year survival rate of 79.7% under outdoor natural conditions (6–33 °C, fluctuating water quality), which is higher than the historical record of the maternal parent LC (≈60%) and comparable to that of WR (81.5%). The survival curve of LWR followed a “convex” pattern, with mortality concentrated in the early juvenile stage, after which survival remained relatively stable. This high survival under natural fluctuating conditions suggests good environmental adaptability, although dedicated stress challenge tests are needed to confirm stress tolerance. These findings indicate that LWR possesses excellent overall survival performance and good long-term survival potential, demonstrating heterosis in survival-related traits. For instance, in clariid catfish, the hybrid *Clarias macromystax* × *C. gariepinus* showed a larval survival rate below 70% [70], whereas the hybrid of Pelteobagrus species achieved a survival rate of 76% at a density of 90,000 individuals per tank [71]. In contrast, the survival rates of the hybrid fish in the present study not only far exceeded the aforementioned cases but were also comparable to the 96.3% survival rate obtained in seawater by the cichlid hybrid of Nile tilapia (*Oreochromis niloticus*) × Mozambique tilapia (*O. mossambicus*) [72]. Moreover, the rearing environment in this study (freshwater) is more representative of practical production conditions, thereby demonstrating positive heterosis. This finding is consistent with the results of an interspecific hybridization study in the genus Tor: the hybrid combination *Tor tambroides* × *T. douronensis* also exhibited significant heterosis in survival rate [61]. Improved survival rates generally reflect enhanced stress resistance and environmental adaptability of hybrid offspring. Systematic observations in common carp hybridization studies indicate that resistance-related traits such as cold tolerance, disease resistance, and survival rate often display characteristics influenced by one parent, with a pronounced maternal effect [73,74,75]. In the present study, the survival rates of the F_1_ hybrids exhibited heterosis, demonstrating that their stress resistance is not simply inherited from a single parent but benefits from the combined effects of both parents. From the perspective of practical application, improved survival rates hold significant economic value. In fish farming, survival during the seed-rearing stage is directly related to aquaculture profitability [76,77]. The F_1_ hybrids in this study maintained high survival rates at both 15 days post-hatching and one year of age, indicating that their strong stress resistance is not only present during the juvenile stage but also persists into the adult stage, conferring strong potential for practical aquaculture application. However, it should be noted that, although the parental and hybrid groups were reared in parallel under the same outdoor conditions, the inherent environmental variability (e.g., temperature fluctuation, light, rainfall, natural food supply) limited the validity of formal statistical comparisons of survival rates. Therefore, the survival data in Table 6 are presented descriptively to show the general trend of LWR survival. Future studies conducted under controlled conditions (e.g., indoor recirculating aquaculture systems) with replicated tanks and standardized environmental parameters will be necessary to test the statistical significance of survival differences between hybrid and parental groups.

## 4. Materials and Methods

### 4.1. Moral Statement

The experimental fish used in this study are not classified as rare or endangered species. All fish were either bred at the Engineering Research Center of Polyploid Fish Reproduction and Breeding of the State Education Ministry, Hunan Normal University, China, or donated by the relevant agricultural law enforcement authorities. Prior to dissection, all fish were anesthetized with 80 mg/L MS-222, and all procedures were conducted in accordance with ethical requirements.

### 4.2. Production and Rearing of Hybrid Offspring

The WR (2n = 100) used in this study were obtained from the Engineering Research Center of Polyploid Fish Reproduction and Breeding of the State Education Ministry, Hunan Normal University, China. Dongting Lake native crucian carp (LC) (2n = 100) were collected from the Dongting Lake water system in Yueyang City, Hunan Province. All LC individuals used in this study were confirmed by flow cytometry to be diploid (2n = 100), with no triploid or tetraploid individuals present. From March to June, 25 mature female LC and 50 male WR were selected. A single synchronized spawning event was induced by hormonal injection (oxytocin analog) on the same day. Artificial insemination was performed using pooled eggs from all females and pooled milt from all males, followed by gentle mixing. All F_1_ hybrid offspring (LWR) came from this single breeding batch. Hormonal injection was administered using a single intraperitoneal injection of LHRH-A_2_ combined with DOM and HCG. Females received 10–15 μg LHRH-A_2_ + 1.5–4 mg DOM + 800–1000 IU HCG per kg body weight, while males received half that dose. Injections were performed at 21:00, and ovulation/spermiation occurred 10–12 h later, at which point artificial insemination was performed. This protocol has been routinely used in our laboratory for Carassius distant hybridization. Fertilized eggs were incubated in three replicate hatching tanks (500 L each, ≈2000 eggs/tank) under 24 h flowing water stimulation using the same system’s flow of water to maintain suitable and same water temperature and water quality parameters. At the same time, water temperature and other water quality parameters were measured using a portable multifunctional water quality analyzer (Lianhua Yongxing Technology Co., Ltd., Beijing, China). When the water quality parameters are inconsistent, the water quality parameters are regulated through the flow system. A total of 2000 randomly selected fertilized eggs were used to calculate the fertilization rate (number of embryos at the blastula stage/total number of eggs × 100%) (The blastula stage was chosen as the endpoint because it represents a stable developmental window after the mid-blastula transition, excluding early pseudo-fertilized or developmentally arrested eggs, and thus provides a more accurate estimate of truly viable embryos.)and the hatching rate (number of hatched embryos/total number of eggs × 100%). After all larvae hatched, they were reared in the hatching tanks for an additional 2–3 days. Once swim bladders were observed, the larvae were transferred to nursery ponds that had been treated with fertilizer paste one week in advance. During the early nursery stage, the larvae were fed with soybean milk, which was gradually replaced with powdered feed and subsequently with pellet feed as the fish developed and grew.

### 4.3. Post-Hybridization Morphology and Fertility

For morphological analysis, 20 one-year-old adult fish were randomly selected from each of the LC, WR, and LWR groups, totaling 60 individuals. Body color was assessed visually by three independent observers under the same natural light conditions (between 10:00 and 14:00 on sunny days). The final description represents the consensus of all three observers. No quantitative colorimeter or standard color chart was used. Body weight ranges were LC 75–85 g, WR 330–340 g, and LWR 290–305 g (Table 5). The measured morphological traits were divided into two categories: morphometric traits (body length, total length, body weight, body height, head length, head height, caudal peduncle length, and caudal peduncle height) and meristic traits (lateral line scales, scales above the lateral line, scales below the lateral line, dorsal fin rays, pelvic fin rays, and anal fin rays). Morphometric traits were measured using electronic digital calipers (0.01 mm) and an electronic balance (0.1 g). Meristic traits were counted under a dissecting microscope. All measurements were performed by the same person. In addition, 10 LWR individuals (separately sampled, not overlapping with the morphological analysis) were randomly selected, and their gonadal tissues were collected for the observation of gonadal development using the paraffin section method [78]. It is worth noting that the LWR individuals (one-year-old adults) used for gonadal histology were sampled in May. The natural breeding season of their parents, LC (which reaches sexual maturity at 1–2 years of age, with fast-growing individuals maturing within the same year) and WR (which reaches sexual maturity at 1 year of age), is from March to June (when the water temperature rises above 15 °C). In this study, mature gametes were obtained from both parental species through artificial induced breeding during this period. All data are presented as mean ± standard deviation (SD). Statistical analyses were performed using SPSS 26.0. For comparisons among the three groups (LC, WR, LWR), one-way analysis of variance (ANOVA) was conducted, followed by Tukey’s honestly significant difference (HSD) post hoc test for pairwise comparisons. For comparisons between two groups (e.g., LWR vs. LC, LWR vs. WR), independent-samples *t*-test was applied. A significance level of *p* < 0.05 was considered statistically significant.

### 4.4. Ploidy Level Examination

The ploidy level of the hybrid progeny (LWR) was analyzed and evaluated by measuring DNA content and chromosome number. Thirty 1-year-old fishes from each group were used for flow cytometric analysis of mean DNA content in LC, WR, and LWR using a flow cytometer (Cell Counter Analyzer, Partec, Münster, Germany). Approximately 0.2 mL of ACD anticoagulant was drawn into a 1 mL sterile syringe, and an appropriate volume of blood was collected from the caudal vein of each experimental fish. Sample processing was performed following previously described methods [12]. Using RCC (2n = 100) as a reference, a Yates-corrected chi-square test was applied to determine whether the mean DNA content measured in LWR deviated from the expected value. In addition, ten fish from each group were selected, and kidney tissues were collected to prepare chromosome slides from renal cells. The preparation method followed that described by Liu et al. with minor modifications: colchicine dose was reduced to 1.5 μg/g body weight (from 2 μg/g) and injected intraperitoneally for 3 h (instead of 2 h); hypotonic treatment was performed using 0.075 M KCl for 30 min (instead of distilled water for 20 min); fixation solution (methanol:acetic acid = 3:1) was changed twice before dropping. For each fish, 30 well-spread metaphase plates were examined under a light microscope (100×) to count chromosome numbers. A fish was identified as diploid (2n = 100) if ≥90% of the counted metaphases (i.e., ≥27 out of 30) showed 100 chromosomes (or 98–102, accounting for technical variation). For DNA content analysis, 30 fish per group were analyzed by flow cytometry. Using red crucian carp (RCC, 2n = 100) as a reference, a sample was classified as diploid if the ratio of its mean DNA content to that of RCC fell within 0.95–1.05.

### 4.5. Genomic DNA Extraction, PCR, and Sequencing

For LC, WR, and LWR, 20 individuals were randomly selected from each group, and tail fin tissue was collected. Total genomic DNA was extracted using the Universal Genomic DNA Extraction Kit (OMEGA, Norcross, GA, USA). Primers for 5S rDNA amplification (F: 5′-GCTATGCCCGATCGCGTCTGA-3′; R:5′-CAGGTTGGTATGGCCGTAAGC-3′) were designed and synthesized based on NCBI sequences. The PCR amplification program was performed according to the method described in a previously published study [35]. Four samples were randomly selected from each fish group for 5S rDNA cloning, and ten positive clones per band from each sample were sequenced, yielding a total of 360 positive clones. One sample from each fish group was sent to Beijing Tsingke Biotech Co., Ltd. for mtDNA sequencing. After sequencing, the results were processed and analyzed using SnapGene (version 8.2.1).

### 4.6. Study on Body Weight, Growth Rate, and Survival Rate

500 juveniles of LC, WR, and LWR, which had been reared in hatching tanks for at least seven days, were randomly selected and transferred to the cement pond (approximately 70 m^2^ per pond) for further rearing. The initial stocking density was 500 fish per pond (approximately 7.1 fish/m^2^). Prior to stocking, an equal amount of fertilizer paste was applied to each pond. The culture conditions in the cement ponds were maintained as similar as possible: dissolved oxygen concentration above 5 mg/L, water temperature maintained between 18 °C and 33 °C, pH controlled within the range of 7.0 to 8.5, and continuous water flow throughout the day. During the nursery stage, each pond received equal amounts of soybean milk daily. As the fish grew, they were fed with formulated feed twice a day, in the morning and evening (feed specifications: crude protein ≥ 30%, crude fat ≥ 3.5%, crude fiber ≤ 12%, crude ash ≤ 15%, total phosphorus ≥ 0.8%, lysine ≥ 1.6%, Yueyang Yumeikang Biotechnology Co., Ltd., Yueyang, China). Pellet size was adjusted dynamically according to the size of the fish. When the experimental fish reached six months of age (juvenile stage) and one year of age (adult stage), the water in each pond was completely drained, and the remaining fish were counted to ensure accuracy of the experimental results. The survival rate at each stage was calculated as (number of remaining fish/500) × 100%. In addition, 20 individuals were randomly selected from each experimental group for body weight measurement. Growth performance was evaluated by measuring body weight (g) at 6 months and 1 year of age. At each time point, 20 fish were randomly selected from each group and weighed using an electronic balance (accuracy 0.1 g). Weight gain percentage from 6 months to 1 year was calculated as [(W_1_ year − W_6_ months)/W_6_ months] × 100% for additional comparison.

## 5. Conclusions

Based on the findings of this study, we conclude that distant hybridization is indeed an effective approach for improving existing fish varieties for aquaculture, thereby promoting the sustainable development of fisheries. However, it should be emphasized that the conservation of native germplasm resources must be based on the protection of wild native populations, not on hybridization. The value of hybridization lies in germplasm improvement and sustainable utilization, not in replacing conservation. Moreover, it can generate traits beneficial to aquaculture economics, particularly rapid growth and enhanced stress tolerance. In summary, we successfully created a novel allodiploid fish (LWR, 2n = 100) through distant hybridization. This hybrid exhibits morphological characteristics intermediate between the two parents, with bisexual fertility, and displays varying degrees of recombination and mutation in both 5S rDNA and mtDNA, with mtDNA following maternal inheritance. Additionally, compared with LC, LWR exhibits superior growth traits and outperforms both parents in terms of survival rate and stress tolerance. By proposing a novel hybrid combination, this study provides valuable materials for improving aquaculture traits of crucian carp, advancing the aquaculture economy, and investigating the genetic characteristics of distant hybrid offspring.

## Figures and Tables

**Table 1:** The countable traits between LC, WR and LWR.

Fish Type	Lateral Line Scales	Upper Lateral Line Scales	Lower Lateral Line Scales	Dorsal Fins	Abdominal Fins	Anal Fins
LC	27–30	5–7	6	III + 16–18	9	III + 6
WR	30–31	6–7	6–7	III + 17–18	8–9	III + 6–7
LWR	28–31	6–7	5–7	III + 16–19	8–9	III + 6–7

**Table 2:** The measurable traits between LC, WR and LWR.

Fish Type	WL/BL	BL/BH	BL/HL	HL/HH	CPL/CPH
LC	1.24 ± 0.03	3.41 ± 0.10	3.83 ± 0.12	1.10 ± 0.02	0.87 ± 0.02
WR	1.24 ± 0.04	2.21 ± 0.13 ^a^	3.73 ± 0.16	1.18 ± 0.07	0.79 ± 0.05
LWR	1.25 ± 0.02	2.35 ± 0.07 ^a^	3.54 ± 0.13 ^a,b^	1.22 ± 0.07 ^a^	0.83 ± 0.05

^a^ indicates a significant difference with LC, *p* < 0.05, ^b^ indicates a significant difference with WR, *p* < 0.05.

**Table 3:** Statistical table of average DNA content of LC, WR and LWR.

Fish Type	Average DNA Content	Ratio	
		Detection Value	Expected Value
RCC	99.85		
LC	99.42	LC/RCC = 0.996 ^a^	1
WR	100.01	WR/RCC = 1.001 ^a^	1
LWR	99.55	LWR/RCC = 0.997 ^a^	1

^a^ indicates no significant difference from the expected value, *p* > 0.05.

**Table 4:** The specific structural features of the mtDNA of LWR.

Start	End	Length	Strand	Gene
1	69	69	+	*trnF(gaa)*
70	1023	954	+	*rrnS*
1024	1095	72	+	*trnV(tac)*
1096	2777	1682	+	*rrnL*
2778	2853	76	+	*trnL2(taa)*
2855	3829	975	+	*ND1*
3834	3905	72	+	*trnI(gat)*
3904	3974	71	−	*trnQ(ttg)*
3976	4044	69	+	*trnM(cat)*
4045	5089	1045	+	*ND2*
5090	5160	71	+	*trnW(tca)*
5163	5231	69	−	*trnA(tgc)*
5233	5305	73	−	*trnN(gtt)*
5308	5339	32	+	rep_origin
5339	5407	69	−	*trnC(gca)*
5407	5477	71	−	*trnY(gta)*
5479	7029	1551	+	*COX1*
7030	7100	71	−	*trnS2(tga)*
7104	7175	72	+	*trnD(gtc)*
7188	7878	691	+	*COX2*
7879	7954	76	+	*trnK(ttt)*
7956	8120	165	+	*ATP8*
8114	8797	684	+	*ATP6*
8797	9581	785	+	*COX3*
9582	9653	72	+	*trnG(tcc)*
9654	10,002	349	+	*ND3*
10,003	10,072	70	+	*trnR(tcg)*
10,073	10,369	297	+	*ND4L*
10,363	11,743	1381	+	*ND4*
11,744	11,812	69	+	*trnH(gtg)*
11,813	11,881	69	+	*trnS1(gct)*
11,883	11,955	73	+	*trnL1(tag)*
11,959	13,782	1824	+	*ND5*
13,779	14,300	522	−	*ND6*
14,301	14,369	69	−	*trnE(ttc)*
14,375	15,515	1141	+	*CYTB*
15,516	15,587	72	+	*trnT(tgt)*
15,587	15,656	70	−	*trnP(tgg)*
15,657	16,580	924	+	D-loop

**Table 5:** Comparison of the mean body weight of LC, WR, LWR (g).

Fish Type	6 Months Old	1 Age
LC	17.7 ± 2.9	79.95 ± 8.2
WR	51.1 ± 1.1	335.5 ± 0.7
LWR	43.4 ± 5.5	297.0 ± 9.2

**Table 6:** Survival rates of LWR at different age groups.

Age	15 Days	1 Months	3 Months	6 Months	1 Year
Survival rate	91.9%	84.6%	81.2%	80.2%	79.7%

## Data Availability

The original contributions presented in this study are included in the article. Further inquiries can be directed to the corresponding author (zyshen@hunnu.edu.cn).

## Supplementary Materials

The following supporting information can be downloaded at: https://www.mdpi.com/article/10.3390/ijms27115049/s1.
