# Supplementary material for: New Hybrid Combination for Local Crucian Carp Germplasm Improvement: Dongting Lake Crucian Carp (♀) × Hefang Crucian Carp (♂)
Source: Int J Mol Sci. 2026 Jun 3;27(11):5049. doi: 10.3390/ijms27115049 (PMC13256677; doi:10.3390/ijms27115049)
Supplement: Supplementary file 1 [file ijms-27-05049-s001.zip › ijms-4305335-supplementary.pdf]

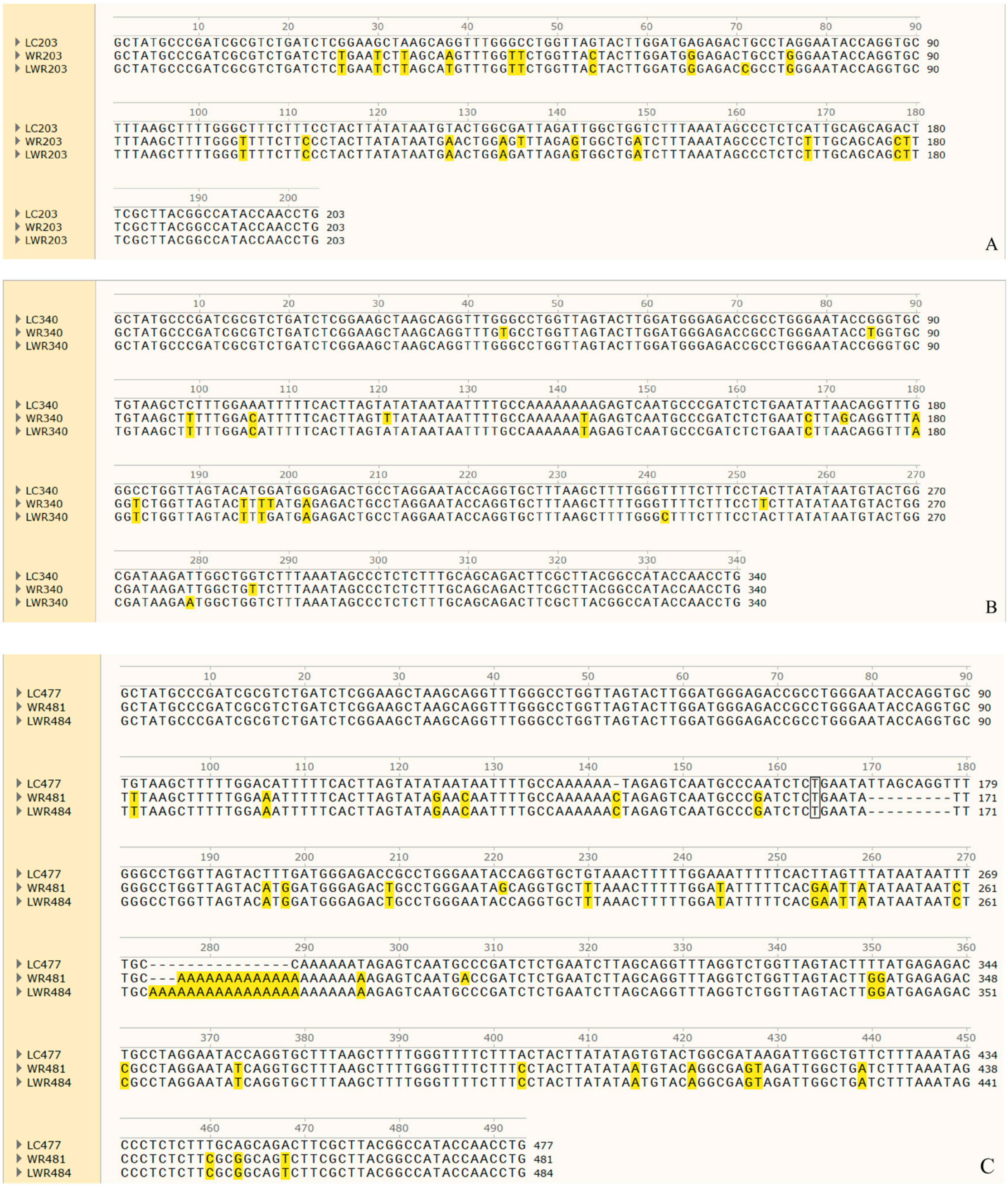

Figure S1. Comparison of 5S rDNA sequences of LC, WR, and LWR (A: 203 bp sequence alignment of LC, WR, and LWR. B: 340 bp sequence alignment of LC, WR, and LWR. C: alignment of LC's 477 bp sequence, WR's 481 bp sequence, and LWR's 484 bp sequence).

```
            10        20        30        40        50        60        70        80
LC   GCTAGCGTAGCTTAATACAAAGCATAGCACTGAAGATGCTAAGATGAGACCTAAAAATCTCCGCATGCACAAAGGCATGG 80
WR   GCTAGCGTAGCTTAATACAAAGCATAGCACTGAAGATGCTAAGATGAGACCTAAAAATCTCCGCATGCACAAAGGCATGG 80
LWR  GCTAGCGTAGCTTAATACAAAGCATAGCACTGAAGATGCTAAGATGAGACCTAAAAATCTCCGCATGCACAAAGGCATGG 80

            90       100       110       120       130       140       150       160
LC   TCCCGACCTTATTATCAGCTCTAACTCAACTTACACATGCAAGTCTCCGCACCCCAGTGAATATGCCCTCAATCCCCCTA 160
WR   TCCCGACCTTATTATCAGCTCTAACTCAACTTACACATGCAAGTCTCCGCACCCCAGTGAATATGCCCTCAATCCCCCTA 160
LWR  TCCCGACCTTATTATCAGCTCTAACTCAACTTACACATGCAAGTCTCCGCACCCCAGTGAATATGCCCTCAATCCCCCTA 160

           170       180       190       200       210       220       230       240
LC   CCCGGGGACGAGGAGCGGGCATCAGGCACAAATATTAGCCCAAGACGCCTAGCCGAGCCACACCCCCAAGGGAATTCAGC 240
WR   CCCGGGGACGAGGAGCGGGCATCAGGCACAAATATTAGCCCAAGACGCCTAGCCGAGCCACACCCCCAAGGGAATTCAGC 240
LWR  CCCGGGGACGAGGAGCGGGCATCAGGCACAAATATTAGCCCAAGACGCCTAGCCGAGCCACACCCCCAAGGGAATTCAGC 240

           250       260       270       280       290       300       310       320
LC   AGTGATAAACATTAAGCCATAAGTGAAAACTTGACTCAGTTAGTGTTAAGAGGGCCGGTAAAACTCGTGCCAGCCACCGC 320
WR   AGTGATAAACATTAAGCCATAAGTGAAAACTTGACTCAGTTAGTGTTAAGAGGGCCGGTAAAACTCGTGCCAGCCACCGC 320
LWR  AGTGATAAACATTAAGCCATAAGTGAAAACTTGACTCAGTTAGTGTTAAGAGGGCCGGTAAAACTCGTGCCAGCCACCGC 320

           330       340       350       360       370       380       390       400
LC   GGTTAGACGAGAGGCCCTAGTTGATATTACAACGGCGTAAAGGGTGGTTAAGGATAAATAAAAATAAAGTCAAATGGCCC 400
WR   GGTTAGACGAGAGGCCCTAGTTGATATTACAACGGCGTAAAGGGTGGTTAAGGATAAATAAAAATAAAGTCAAATGGCCC 400
LWR  GGTTAGACGAGAGGCCCTAGTTGATATTACAACGGCGTAAAGGGTGGTTAAGGATAAATAAAAATAAAGTCAAATGGCCC 400

           410       420       430       440       450       460       470       480
LC   CTTGGCCGTCATACGCTTCTAGGCGTCCGAAGCCCTAATACGAAAGTAACTTTAATGAACCCACCTGACCCCACGAAAGC 480
WR   CTTGGCCGTCATACGCTTCTAGGCGTCCGAAGCCCTAATACGAAAGTAACTTTAATGAACCCACCTGACCCCACGAAAGC 480
LWR  CTTGGCCGTCATACGCTTCTAGGCGTCCGAAGCCCTAATACGAAAGTAACTTTAATGAACCCACCTGACCCCACGAAAGC 480

           490       500       510       520       530       540       550       560
LC   TGAGGAACAAACTGGGATTAGATACCCCACTATGCTCAGCCGTAAACTTAGACATCCAACTACAATAGATGTCCGCCAGG 560
WR   TGAGGAACAAACTGGGATTAGATACCCCACTATGCTCAGCCGTAAACTTAGACATCCAACTACAATAGATGTCCGCCAGG 560
LWR  TGAGGAACAAACTGGGATTAGATACCCCACTATGCTCAGCCGTAAACTTAGACATCCAACTACAATAGATGTCCGCCAGG 560

           570       580       590       600       610       620       630       640
LC   GTACTACGAGCATTAGCTTAAAACCCAAAGGACCTGACGGTGTCTCAGATCCCCCTAGAGGAGCCTGTTCTAGAACCGAT 640
WR   GTACTACGAGCATTAGCTTAAAACCCAAAGGACCTGACGGTGTCTCAGACCCCCCTAGAGGAGCCTGTTCTAGAACCGAT 640
LWR  GTACTACGAGCATTAGCTTAAAACCCAAAGGACCTGACGGTGTCTCAGATCCCCCTAGAGGAGCCTGTTCTAGAACCGAT 640

           650       660       670       680       690       700       710       720
LC   AACCCCCGTTCAACCTCACCACTTCTAGCCAACCCAGCCTATATACCGCCGTCGTCAGCTTACCCTGTGAAGGTAATAAA 720
WR   AACCCCCGTTCAACCTCACCACTTCTAGCCAACCCAGCCTATATACCGCCGTCGTCAGCTTACCCTGTGAAGGTAATAAA 720
LWR  AACCCCCGTTCAACCTCACCACTTCTAGCCAACCCAGCCTATATACCGCCGTCGTCAGCTTACCCTGTGAAGGTAATAAA 720

           730       740       750       760       770       780       790       800
LC   AGTAAGCAAAATGGGTACAACCCAAAACGTCAGGTCGAGGTGTAGCGCATGGAGTGGGAAGAAATGGGCTACATTTTCTA 800
WR   AGTAAGCAAAATGGGTACAACCCAAAACGTCAGGTCGAGGTGTAGCGCATGAGGTGGGAAGAAATGGGCTACATTTTCTA 800
LWR  AGTAAGCAAAATGGGTACAACCCAAAACGTCAGGTCGAGGTGTAGCGCATGAGGTGGGAAGAAATGGGCTACATTTTCTA 800

           810       820       830       840       850       860       870       880
LC   ACATAGAATATTACGAACATGCACCATGAAACAGTGCTTGAAGGAGGATTTAGTAGTAAAAGGGAAATAGAGTGTCCCTT 880
WR   ACATAGAATATTACGAATATGCACCATGAAACAGTGCTTGAAGGAGGATTTAGTAGTAAAAGGGAAATAGAGTGTCCCTT 880
LWR  ACATAGAATATTACGAATATGCACCATGAAACAGTGCTTGAAGGAGGATTTAGTAGTAAAAGGGAAATAGAGTGTCCCTT 880

           890       900       910       920       930       940       950       960
LC   TGAACCCGGCTCTGAGACGCGTACACACCGCCCGTCACTCTCCCCTGTCAAAATGCACCAAAAATATCTAATATAATAGC 960
WR   TGAACCCGGCTCTGAGACGCGTACACACCGCCCGTCACTCTCCCCTGTCAAAATGCACCAAAAATATCTAATATAATAGC 960
LWR  TGAACCCGGCTCTGAGACGCGTACACACCGCCCGTCACTCTCCCCTGTCAAAATGCACCAAAAATATCTAATATAATAGC 960
```

```
           970       980       990       1000      1010      1020      1030      1040
LC   ACTGACAAGGGGAGGCAAGTCGTAACACGGTAAGTGTACCGGAAGGTGCACTTGGATCAAACCCAGGGTGTGGCTGAGTT 1040
WR   ACTGACAAGGGGAGGCAAGTCGTAACACGGTAAGTGTACCGGAAGGTGCACTTGGATCAAACCCAGGGTGTGGCTGAGTT 1040
LWR  ACTGACAAGGGGAGGCAAGTCGTAACACGGTAAGTGTACCGGAAGGTGCACTTGGATCAAACCCAGGGTGTGGCTGAGTT 1040

           1050      1060      1070      1080      1090      1100      1110      1120
LC   AGTCAAGCATCTCACTTACACCGAGAAGACATCCATGCAAGTTGGATCGCCCTGAGCCAAACAGCTAGCTTAACTACCTA 1120
WR   AGTCAAGCATCTCACTTACACCGAGAAGACATCCATGCAAGTTGGATCGCCCTGAGCCAAACAGCTAGCTTAACTACCTA 1120
LWR  AGTCAAGCATCTCACTTACACCGAGAAGACATCCATGCAAGTTGGATCGCCCTGAGCCAAACAGCTAGCTTAACTACCTA 1120

           1130      1140      1150      1160      1170      1180      1190      1200
LC   ATAACTAAACAATATAAATAAAATAAGATAGACCTAACACTAAAAATTAAATCATTCTTTTACCTGAGTATGGGCGACAG 1200
WR   ATAACTAAACAATATAAATAAAATAAGATAGACCTAACACTAAAAATTAAATCATTCTTTTACCTGAGTATGGGCGACAG 1200
LWR  ATAACTAAACAATATAAATAAAATAAGATAGACCTAACACTAAAAATTAAATCATTCTTTTACCTGAGTATGGGCGACAG 1200

           1210      1220      1230      1240      1250      1260      1270      1280
LC   AAAAGGTTCCACAAAGCGATAGAAATAGTACCGCAAGGGAAAGCTGAAAGAGAAATGAAATAACCCATATAAGCAATAAA 1280
WR   AAAAGGTTCCACAAAGCAATAGAAATAGTACCGCAAGGGAAAGCTGAAAGAGAAATGAAATAACCCATATAAGCAATAAA 1280
LWR  AAAAGGTTCCACAAAGCGATAGAAATAGTACCGCAAGGGAAAGCTGAAAGAGAAATGAAATAACCCATATAAGCAATAAA 1280

           1290      1300      1310      1320      1330      1340      1350      1360
LC   AAGCAAAGATTAAACCTTGTACCTTTTGCATCATGATTTAGCCAGTACACCCAAGCAAAGAGACCTTTAGTTTGAAACCC 1360
WR   AAGCAAAGATTAAACCTTGTACCTTTTGCATCATGATTTAGCCAGTACACCCAAGCAAAGAGACCTTTAGTTTGAAACCC 1360
LWR  AAGCAAAGATTAAACCTTGTACCTTTTGCATCATGATTTAGCCAGTACACCCAAGCAAAGAGACCTTTAGTTTGAAACCC 1360

           1370      1380      1390      1400      1410      1420      1430      1440
LC   CGAAACCAGGTGAGCTACCCCGAGACAGCCTATTGAGGGCCAACCCGTCTCTGTGGCAAAAGAGTGGGAAGAGCTCCGGG 1440
WR   CGAAACCAGGTGAGCTACCCCGAGACAGCCTATTGAGGGCCAACCCGTCTCTGTGGCAAAAGAGTGGGAAGAGCTCCGGG 1440
LWR  CGAAACCAGGTGAGCTACCCCGAGACAGCCTATTGAGGGCCAACCCGTCTCTGTGGCAAAAGAGTGGGAAGAGCTCCGGG 1440
```

```
           1450      1460      1470      1480      1490      1500      1510      1520
LC   TAGAAGTGACAGACCTACCGAACCTGGTGATAGCTGGTTGCCTAAGAAATGGATAGAAGTTCAGCCTCGTACTCCCCAAA 1520
WR   TAGAAGTGACAGACCTACCGAACCTGGTGATAGCTGGTTGCCTAAGAAATGGATAGAAGTTCAGCCTCGTACTCCCCAAA 1520
LWR  TAGAAGTGACAGACCTACCGAACCTGGTGATAGCTGGTTGCCTAAGAAATGGATAGAAGTTCAGCCTCGTACTCCCCAAA 1520

           1530      1540      1550      1560      1570      1580      1590      1600
LC   TCAAATAAACATTAATAAGACAACAAGAGAAACATACGAGAGTTAGTTAAAGGGGGTACAGCCCCTTTGACAAAGGATAC 1600
WR   TCAAATAAACATCAATAAGACAACAAGAGAAACATACGAGAGTTAGTTAAAGGGGGTACAGCCCCTTTGACAAAGGATAC 1600
LWR  TCAAATAAACATCAATAAGACAACAAGAGAAACATACGAGAGTTAGTTAAAGGGGGTACAGCCCCTTTGACAAAGGATAC 1600

           1610      1620      1630      1640      1650      1660      1670      1680
LC   AACCTTTCTAGGAGGATAAAGATCATAATACATAAAACATACTGTTCTAGTGGGCCTAAAAGCAGCCACCTAGATAGAAA 1680
WR   AACCTTTCTAGGAGGATAAAGATCATAATTCATAAAACATACTGTTCTAGTGGGCCTAAAAGCAGCCACCTAGATAGAAA 1680
LWR  AACCTTTCTAGGAGGATAAAGATCATAATTCATAAAACATACTGTTCTAGTGGGCCTAAAAGCAGCCACCTAGATAGAAA 1680

           1690      1700      1710      1720      1730      1740      1750      1760
LC   GCGTTAAAGCTCAGACAGATAGAAGTTTATTATCCTGATAATATATCTTACTCCCCTAAATACTATTAGGCCAACCCATG 1760
WR   GCGTTAAAGCTCAGACAGATAGAAGTTTATTATCCTGATAATATATCTTACTCCCCTAAATACTATTAGGCCAACCCATG 1760
LWR  GCGTTAAAGCTCAGACAGATAGAAGTTTATTATCCTGATAATATATCTTACTCCCCTAAATACTATTAGGCCAACCCATG 1760

           1770      1780      1790      1800      1810      1820      1830      1840
LC   CCCACATGGAAGAGATTATGCTAAAATGAGTAACAAGAAGGCCCGCCCTTCTCCAAGCACAAGTGTAAGCCAAATCGGAC 1840
WR   CCCACATGGAAGAGATTATGCTAAAATGAGTAACAAGAAGGCCCGCCCTTCTCCAAGCACAAGTGTAAGCCAAATCGGAC 1840
LWR  CCCACATGGAAGAGATTATGCTAAAATGAGTAACAAGAAGGCCCGCCCTTCTCCAAGCACAAGTGTAAGCCAAATCGGAC 1840

           1850      1860      1870      1880      1890      1900      1910      1920
LC   AAGCCATTGGCAACTAACGAACTCAACCAAAGAGAGCAATGTGGTATCACAAAAAAACCTAGAAAAACCCACAACCTAAA 1920
WR   AAGCCATTGGCAACTAACGAACTCAACCAAAGAGAGCAATGTGGTGTCACAAAAAAACCTAGAAAAACCCACAACCCAAA 1920
LWR  AAGCCATTGGCAACTAACGAACTCAACCAAAGAGAGCAATGTGGTATCACAAAAAAACCTAGAAAAACCCACAACCTAAA 1920
```

```
            1930      1940      1950      1960      1970      1980      1990      2000
LC   TATCGTTACCCCCACACTGGAGTGCAACAAAGGAAAGACTAAAAGAAAAGGAAGGAACTCGGCAAACACAAGCCTCGCCT 2000
WR   TATCGTTACCCCCACACTGGAGTGCAACAAAGGAAAGACTAAAAGAAAAGGAAGGAACTCGGCAAACACAAGCCTCGCCT 2000
LWR  TATCGTTACCCCCACACTGGAGTGCAACAAAGGAAAGACTAAAAGAAAAGGAAGGAACTCGGCAAACACAAGCCTCGCCT 2000

            2010      2020      2030      2040      2050      2060      2070      2080
LC   GTTTACCAAAAACATCGCCTCCTGCAACACAACCAAGTATAGGAGGTCCAGCCTGCCCAGTGACTACAAGTTCAACGGCC 2080
WR   GTTTACCAAAAACATCGCCTCCTGCAACACAACCAAGTATAGGAGGTCCAGCCTGCCCAGTGACTACAAGTTCAACGGCC 2080
LWR  GTTTACCAAAAACATCGCCTCCTGCAACACAACCAAGTATAGGAGGTCCAGCCTGCCCAGTGACTACAAGTTCAACGGCC 2080

            2090      2100      2110      2120      2130      2140      2150      2160
LC   GCGGTATTTTGACCGTGCAAAGGTAGCGCAATCACTTGTCTTTTAAATAGAGACCTGTATGAATGGCTAAACGAGGGCTT 2160
WR   GCGGTATTTTGACCGTGCAAAGGTAGCGCAATCACTTGTCTTTTAAATAGAGACCTGTATGAATGGCTAAACGAGGGCTT 2160
LWR  GCGGTATTTTGACCGTGCAAAGGTAGCGCAATCACTTGTCTTTTAAATAGAGACCTGTATGAATGGCTAAACGAGGGCTT 2160

            2170      2180      2190      2200      2210      2220      2230      2240
LC   AACTGTCTCCCCTTTCCAGTCAGTGAAATTGATCTACCCGTGCAGAAGCGGGTATAATAATACAAGACGAGAAGACCCTT 2240
WR   AACTGTCTCCCCTTTCCAGTCAGTGAAATTGATCTACCCGTGCAGAAGCGGGTATAATAATACAAGACGAGAAGACCCTT 2240
LWR  AACTGTCTCCCCTTTCCAGTCAGTGAAATTGATCTACCCGTGCAGAAGCGGGTATAATAATACAAGACGAGAAGACCCTT 2240

            2250      2260      2270      2280      2290      2300      2310      2320
LC   TGGAGCTTAAGGTACAAAACTCAACCACGTTAAGCAACTCAATAAAAAGTGAAAACTTTGTGGAACATGAGATTTTACCT 2320
WR   TGGAGCTTAAGGTACAAAACTCAACCACGTTAAGCAACTCAATAAAAAGTGAAAACTTTGTGGAACATGAGATTTTACCT 2320
LWR  TGGAGCTTAAGGTACAAAACTCAACCACGTTAAGCAACTCAATAAAAAGTGAAAACTTTGTGGAACATGAGATTTTACCT 2320

            2330      2340      2350      2360      2370      2380      2390      2400
LC   TCGGTTGGGGCGACCACGGAGGAAAAAAAAGCCTCCAGGTGGAACGGGAGAATTTCCTAAAACTAAGAGAGACATCTCTA 2400
WR   TCGGTTGGGGCGACCACGGAGGAAAAAAAAGCCTCCAGGTGGAACGGGAGAATTTCCTAAAACTAAGAGAGACATCTCTA 2400
LWR  TCGGTTGGGGCGACCACGGAGGAAAAAAAAGCCTCCAGGTGGAACGGGAGAATTTCCTAAAACTAAGAGAGACATCTCTA 2400
```

```
            2410      2420      2430      2440      2450      2460      2470      2480
LC   AGCCACAGAACATCTGACCAAATATGATCCGGCTGATACAAGCCGATCAACGAACCAAGTTACCCTAGGGATAACAGCGC 2480
WR   AGCCACAGAACATCTGACCAAATATGATCCGGCTAATACAAGCCGATCAACGAACCAAGTTACCCTAGGGATAACAGCGC 2480
LWR  AGCCACAGAACATCTGACCAAATATGATCCGGCTGATACAAGCCGATCAACGAACCAAGTTACCCTAGGGATAACAGCGC 2480

            2490      2500      2510      2520      2530      2540      2550      2560
LC   AATCCTCTCCCAGAGTCCATATCGACGAGGGGGTTTACGACCTCGATGTTGGATCAGGACATCCTAATGGTGCAGCCGCT 2560
WR   AATCCTCTCCCAGAGTCCATATCGACGAGGGGGTTTACGACCTCGATGTTGGATCAGGACATCCTAATGGTGCAGCCGCT 2560
LWR  AATCCTCTCCCAGAGTCCATATCGACGAGGGGGTTTACGACCTCGATGTTGGATCAGGACATCCTAATGGTGCAGCCGCT 2560

            2570      2580      2590      2600      2610      2620      2630      2640
LC   ATTAAGGGTTCGTTTGTTCAACGATTAAAGTCCTACGTGATCTGAGTTCAGACCGGAGCAATCCAGGTCAGTTTCTATCT 2640
WR   ATTAAGGGTTCGTTTGTTCAACGATTAAAGTCCTACGTGATCTGAGTTCAGACCGGAGCAATCCAGGTCAGTTTCTATCT 2640
LWR  ATTAAGGGTTCGTTTGTTCAACGATTAAAGTCCTACGTGATCTGAGTTCAGACCGGAGCAATCCAGGTCAGTTTCTATCT 2640

            2650      2660      2670      2680      2690      2700      2710      2720
LC   GTAACGCTACTTTTCCTAGTACGAAAGGATCGGAAAAGAGGGGCCAATGCTCAAGGCACGCCCCACCCCTAATTTATGAA 2720
WR   GTAACGCTACTTTTCCTAGTACGAAAGGATCGGAAAAGAGGGGCCAATGCTCGAGGCACGCCCCACCCCTAATTTATGAA 2720
LWR  GTAACGCTACTTTTCCTAGTACGAAAGGATCGGAAAAGAGGGGCCAATGCTCAAGGCACGCCCCACCCCTAATTTATGAA 2720

            2730      2740      2750      2760      2770      2780      2790      2800
LC   AACAAATAAATAAAATAAAGGGAGGGCCAAAACCCCAGCTGGCCAAAATAAGGACATACTGGGGTGGCAGAGCATGGTAA 2800
WR   AACAAATAAATAAAATAAAGGGAGGGCCAAGACCCCAGCTGGCCAAAATAAGGACATACTGGGGTGGCAGAGCATGGTAA 2800
LWR  AACAAATAAATAAAATAAAGGGAGGGCCAAGACCCCAGCTGGCCAAAATAAGGACATACTGGGGTGGCAGAGCATGGTAA 2800

            2810      2820      2830      2840      2850      2860      2870      2880
LC   ATTGCGAAAGGCCTAAGCCCTTTTAACCAGAGGTTCAAATCCTCTTCCCAGTTTATGCTAAACACCCTAATAACCCACCT 2880
WR   ATTGCGAAAGGCCTAAGCCCTTTTAACCAGAGGTTCAAATCCTCTTCCCAGTTTATGCTAAACACCCTAATAACCCACCT 2880
LWR  ATTGCGAAAGGCCTAAGCCCTTTTAACCAGAGGTTCAAATCCTCTTCCCAGTTTATGCTAAACACCCTAATAACCCACCT 2880
```

```
            2890      2900      2910      2920      2930      2940      2950      2960
LC   AATTAACCCCTTAGCCTACATCGTACCCGTACTTTTAGCAGTAGCCTTCCTAACACTTATTGAACGAAAGGTATTAGGAT 2960
WR   AATTAACCCCCTAGCCTACATCGTACCCGTACTTTTAGCAGTAGCCTTCCTAACACTTATTGAACGAAAGGTATTAGGAT 2960
LWR  AATTAACCCCTTAGCCTACATCGTACCCGTACTTCTAGCAGTAGCCTTCCTAACACTTATTGAACGAAAGGTATTAGGAT 2960

            2970      2980      2990      3000      3010      3020      3030      3040
LC   ACATGCAACTACGAAAGGGACCAAACGTGGTAGGCCCATACGGATTACTACAACCAATTGCTGACGGAGTAAAACTCTTC 3040
WR   ACATGCAACTACGAAAGGGACCAAACGTGGTAGGCCCATACGGATTACTACAACCAATTGCTGACGGGGTAAAACTCTTC 3040
LWR  ACATGCAACTACGAAAGGGACCAAACGTGGTAGGCCCATACGGATTACTACAACCAATTGCTGACGGAGTAAAACTCTTC 3040

            3050      3060      3070      3080      3090      3100      3110      3120
LC   ATTAAAGAACCCGTCCGCCCATCTACATCCTCCCCATTTCTGTTTTTAGCTGCTCCAGTACTAGCACTAACCCTAGCCAT 3120
WR   ATTAAAGAACCCGTCCGCCCATCTACATCCTCCCCATTTCTGTTTTTAGCTGCTCCAGTACTAGCACTAACCCTAGCCAT 3120
LWR  ATTAAAGAACCCGTCCGCCCATCTACATCCTCCCCATTTCTGTTTTTAGCTGCTCCAGTACTAGCACTAACCCTAGCCAT 3120

            3130      3140      3150      3160      3170      3180      3190      3200
LC   GACCTTATGAGCACCAATACCTATACCTCACCCAGTAACTGATCTTAACCTAGGGATCCTCTTTATTCTGGCCCTATCAA 3200
WR   GACCTTATGAGCACCAATACCTATACCTCACCCAGTAACTGATCTTAACCTGGGGATCCTCTTTATTCTGGCCCTATCAA 3200
LWR  GACCTTATGAGCACCAATACCTATACCTCACCCAGTAACTGATCTTAACCTGGGGATCCTCTTTATTCTGGCCCTATCAA 3200

            3210      3220      3230      3240      3250      3260      3270      3280
LC   GCCTTGCAGTATACTCAATCCTAGGATCAGGATGAGCATCAAATTCGAAATACGCCCTAATTGGAGCACTACGAGCCGTA 3280
WR   GCCTTGCAGTATACTCAATCCTGGGGTCAGGATGAGCATCAAATTCGAAATACGCCCTAATTGGAGCACTACGAGCCGTA 3280
LWR  GCCTTGCAGTATACTCAATCCTGGGGTCAGGATGAGCATCAAATTCGAAATACGCCCTAATTGGAGCACTACGAGCCGTA 3280

            3290      3300      3310      3320      3330      3340      3350      3360
LC   GCCCAAACGATCTCATATGAAGTCAGCCTAGGACTTATCCTTTTATCCGTTATTATTTTCTCCGGGGGATATACACTACA 3360
WR   GCCCAAACGATCTCATATGAGGTCAGCTTAGGACTTATCCTTTTATCCGTTATTATTTTCTCCGGGGGGTATACACTACA 3360
LWR  GCCCAAACGATCTCATATGAAGTCAGCTTAGGACTTATCCTTTTATCCGTTATTATTTTCTCCGGGGGATATACACTACA 3360
```

```
            3370      3380      3390      3400      3410      3420      3430      3440
LC   AACATTTAATACCACTCAGGAAAGCATCTGATTACTAATTCCCGCCTGGCCTTTAGCCGCAATATGGTATATCTCAACAC 3440
WR   AACATTTAATACTACTCAGGAAAGCATCTGATTACTAATTCCCGCCTGGCCTTTAGCCGCAATATGGTATATTTCAACAC 3440
LWR  AACATTTAATACTACTCAGGAAAGCATCTGATTACTAATTCCCGCCTGGCCTTTAGCCGCAATATGGTATATCTCAACAC 3440

            3450      3460      3470      3480      3490      3500      3510      3520
LC   TAGCCGAAACAAACCGAGCACCATTCGACCTAACAGAAGGAGAATCTGAACTAGTGTCCGGCTTTAACGTAGAATATGCA 3520
WR   TAGCCGAGACAAACCGAGCACCATTCGACCTAACAGAAGGGGAATCTGAACTAGTGTCCGGCTTTAACGTAGAATATGCA 3520
LWR  TAGCCGAGACAAACCGAGCACCATTCGACCTAACAGAAGGAGAATCTGAACTAGTGTCCGGCTCTAACGTAGAATATGCA 3520

            3530      3540      3550      3560      3570      3580      3590      3600
LC   GGAGGACCATTTGCACTTTTCTTTCTAGCCGAATACGCCAACATCCTTCTAATAAACACCCTCTCAGCTGTCTTATTTTT 3600
WR   GGAGGACCATTTGCACTTTTCTTTCTAGCCGAATACGCCAACATTCTTCTAATGAACACCCTCTCAGCTGTCTTATTTTT 3600
LWR  GGAGGACCATTTGCACTTTTCTTTCTAGCCGAATACGCCAACATTCTTCTAATGAACACCCTCTCAGCTGTCTTATTTTT 3600

            3610      3620      3630      3640      3650      3660      3670      3680
LC   AGGAGCCTCACACATCCCAAACATACCAGAACTTACAACAGTCAACCTCATAACTAAAGCTGCACTTCTATCCATTCTAT 3680
WR   AGGAGCCTCACACATCCCAAACATACCAGAACTTACAACAATTAACCTCATAACTAAAGCTGCACTACTATCCATTCTAT 3680
LWR  AGGAGCCTCACACATCCCAAACATACCAGAACTTACAACAATTAACCTCATAACTAAAGCTGCACTTCTATCCATTCTAT 3680

            3690      3700      3710      3720      3730      3740      3750      3760
LC   TCCTATGAGTACGAGCCTCGTACCCACGATTCCGTTATGACCAACTAATACACCTAGTATGAAAAAATTTCCTCCCTCTC 3760
WR   TCCTATGAGTACGAGCCTCATACCCACGATTCCGTTATGACCAACTAATACACCTAGTATGAAAAAATTTCCTCCCTCTC 3760
LWR  TCCTATGAGTACGAGCCTCGTACCCACGATTCCGTTATGACCAACTAATACACCTAGTATGAAAAAATTTCCTCCCTCTC 3760

            3770      3780      3790      3800      3810      3820      3830      3840
LC   ACACTAGCCTTTGTACTATGACATACCGCTCTGCCAATTGCACTAGCGGGGCTCCCCCCACAACTATAACTAAGGAACTG 3840
WR   ACACTAGCCTTTGTACTATGACATACCGCTCTGCCAATTGCACTAGCGGGGCTCCCCCCACAACTATAACTAAGGAACTG 3840
LWR  ACACTAGCCTTTGTACTATGACATACCGCTCTGCCAATTGCACTAGCGGGGCTCCCCCCACAACTATAACTAAGGAACTG 3840
```

```
              3850      3860      3870      3880      3890      3900      3910      3920
LC    TGCCTGAGCACCCAAGGACCACTTTGATAGAGTGAATTACAGGGGTTAAAATCCCCTCAGTTCCTAGAAAGAAGGGAATT 3920
WR    TGCCTGAGCACCCAAGGACCACTTTGATAGAGTGAATTACAGGGGTTAAAATCCCCTCAGTTCCTAGAAAGAAGGGAATT 3920
LWR   TGCCTGAGCACCCAAGGACCACTTTGATAGAGTGAATTACAGGGGTTAAAATCCCCTCAGTTCCTAGAAAGAAGGGAATT 3920

              3930      3940      3950      3960      3970      3980      3990      4000
LC    GAACCCATACTCAGGAGATCAAAACTCCTGGTGCTTCCTTTACACCACTTTCTAAGGCGGGGTCAGCTAATGAAGCTTTC 4000
WR    GAACCCATACTCAAGAGATCAAAACTCCTGGTGCTTCCTTTACACCACTTTCTAAGGCGGGGTCAGCTAATAAAGCTTTC 4000
LWR   GAACCCATACTCAGGAGATCAAAACTCCTGGTGCTTCCTTTACACCACTTTCTAAGGCGGGGTCAGCTAATGAAGCTTTC 4000

              4010      4020      4030      4040      4050      4060      4070      4080
LC    GGGCCCATACCCCGAACATGACGGTTAAAGTCCCTCCTCCGCCAATGAACCCATACGTACTTATAATTTTACTATCCAGC 4080
WR    GGGCCCATACCCCGAACATGACGGTTAAAGTCCCTCCTCCGCCAATGAACCCATACGTACTTATAATTTTACTATCCAGT 4080
LWR   GGACCCATACCCCGAACATGACGGTTAAAGTCCCTCCTCCGCCAATGAACCCATACGTACTTATAATTTTACTATCCAGC 4080

              4090      4100      4110      4120      4130      4140      4150      4160
LC    CTAGGACTAGGAACTACTCTAACCTTCGCCAGCTCTCACTGACTTCTAGCTTGAATAGGCCTAGAAATTAACACACTAGC 4160
WR    CTAGGACTAGGAACTACTCTAACCTTCGCCAGCTCTCACTGACTTCTAGCTTGAATAGGCCTAGAAATTAACACACTAGC 4160
LWR   CTAGGACTAGGAACTACTCTAACCTTCGCCAGCTCTCACTGACTTCTAGCTTGAATAGGCCTAGAAATTAACACACTAGC 4160

              4170      4180      4190      4200      4210      4220      4230      4240
LC    AATTACCCCCCTAATAGCACAACACCACCACCCCCGCGCAGTAGAAGCAACAACAAAATACTTCTTAACCCAAGCCACTG 4240
WR    AATTACCCCCCTAATAGCACAACACCACCACCCCCGCGCAGTAGAAGCAACAACAAAATACTTCTTAACCCAAGCCACTG 4240
LWR   AATTACCCCCCTAATAGCACAACACCACCACCCCCGCGCAGTAGAAGCAACAACAAAATACTTCTTAACCCAAGCCACTG 4240

              4250      4260      4270      4280      4290      4300      4310      4320
LC    CAGCAGCAATAATTCTATTTGCCAGCACAACAAATGCCTGAATAACAGGAGAATGGAACATCACCGACCTATCAGACCCC 4320
WR    CAGCAGCAATAATTCTATTTGCCAGCACAACAAATGCCTGAATAACAGGAGAATGGAACATCACCGACCTATCAGACCCC 4320
LWR   CAGCAGCAATAATTCTATTTGCCAGCACAACAAATGCCTGAATAACAGGAGAATGGAACATCACCGACCTATCAGACCCC 4320
```

```
              4330      4340      4350      4360      4370      4380      4390      4400
LC    CTCGCTAACACAATATTTATAACCGCCCTAGCACTTAAGATTGGACTTGCACCAATACACTTCTGAATGCCCGAAGTTAT 4400
WR    CTCGCTAATACAATATTTATAACCGCCCTAGCACTTAAGATTGGACTTGCACCAATACACTTCTGAATGCCCGAAGTTAT 4400
LWR   CTCGCTAACACAATATTTATAACCGCCCTAGCACTTAAGATTGGACTTGCACCAATACACTTCTGAATGCCCGAAGTTAT 4400

              4410      4420      4430      4440      4450      4460      4470      4480
LC    ACAAGGATTAGATCTATTAACAGGACTTATTCTCTCCACATGACAAAAACTTGCCCCCTTCGCACTTATCATTCAAACAG 4480
WR    ACAAGGATTAGATCTATTAACAGGACTTATTCTCTCCACATGACAAAAACTTGCCCCCTTCGCACTCATCATTCAAACAG 4480
LWR   ACAAGGATTAGATCTATTAACAGGACTTATTCTCTCCACATGACAAAAACTTGCCCCCTTCGCACTTATCATTCAAACAG 4480

              4490      4500      4510      4520      4530      4540      4550      4560
LC    CACAAAACATTGACCCGCTACTATTAACACTACTAGGGGTAACATCTACATTAGTGGGCGGGTGAGGAGGCCTGAACCAA 4560
WR    CACAAAACATTGACCCACTACTATTAACATTACTAGGGGTAACATCCACATTAGTAGGCGGGTGAGGAGGCCTAAACCAA 4560
LWR   CACAAAACATTGACCCGCTACTATTAACACTACTAGGGGTAACATCTACATTAGTGGGCGGGTGAGGAGGCCTGAACCAA 4560

              4570      4580      4590      4600      4610      4620      4630      4640
LC    ACCCAGCTACGAAAAATTCTAGCCTACTCCTCAATTGCACACATAGGATGAATAATTATTGTAATCCAATACGCCCCCCA 4640
WR    ACCCAGCTACGAAAAATCCTAGCCTACTCCTCAATTGCACACATAGGGTGAATAATTATTGTAATTCAATACGCCCCCCA 4640
LWR   ACCCAGCTACGAAAAATCCTAGCCTACTCCTCAATTGCACACATAGGATGAATAATTATTGTAATCCAATACGCCCCCCA 4640

              4650      4660      4670      4680      4690      4700      4710      4720
LC    GCTCACCCTGATTGCACTAGGGACATACATTATCATGACCTCCGCAGCATTCCTAACCCTAAAAATATCACTGACAACTA 4720
WR    ACTCACCCTAATTGCACTAGGGACATACATTGTCATGACCTCCGCAGCATTCCTAACCCTAAAAATATCACTGACAACTA 4720
LWR   GCTCACCCTAATTGCACTAGGGACATACATTATCATGACCTCCGCAGCATTCCTAACCCTAAAAATATCACTGACAACTA 4720

              4730      4740      4750      4760      4770      4780      4790      4800
LC    AACTCAGCACGCTAGCAACAACCTGATCAAAAAGCCCCATCCTAACGGCAACAACTGCCCTAGTATTATTATCACTAGGC 4800
WR    AACTCAGCACGCTAGCAACAACCTGATCAAAAAACCCCATCCTAACAGCAACAACTGCCCTGGTATTATTATCACTAGGC 4800
LWR   AACTCAGCACGCTAGCAACAACCTGATCAAAAAGCCCCATCCTAACGGCAACAACTGCCCTAGTATTATTATCACTAGGC 4800
```

```
             4810      4820      4830      4840      4850      4860      4870      4880
LC   GGCCTACCGCCGCTCACAGGGTTTATACCAAAATGATTAATTTTACAAGAACTGACAAAACAAGATCTCCCCATTATCGC 4880
WR   GGCCTCCCGCCGCTCACAGGGTTTATACCAAAATGATTAATTTTACAAGAACTGACAAAGCAAGATCTCCCCATTATCGC 4880
LWR  GGCCTACCGCCGCTCACAGGGTTTATACCAAAATGATTAATTTTACAAGAACTGACAAAACAAGATCTCCCCATTATCGC 4880

             4890      4900      4910      4920      4930      4940      4950      4960
LC   CACAATCATAGCCCTAGCCGCCCTAATTAGCCTATACTTCTATCTACGACTATGCTACGCGATAACATTAACCATCTCCC 4960
WR   CACAATTATAGCCCTAGCCGCCCTAATTAGCCTATACTTCTATCTACGACTATGCTACGCGATAACATTAACCATCTCCC 4960
LWR  CACAATCATAGCCCTAGCCGCCCTAATTAGCCTATACTTCTATCTACGACTATGCTACGCGATAACATTAACCATCTCCC 4960

             4970      4980      4990      5000      5010      5020      5030      5040
LC   CCAATACAACCAACTCAACCACCCCCTGACGAACCCAAACAACCCAAACCTCCATACCTCTAGCCCTATTTACCATAGCC 5040
WR   CCAATACAACCAACTCAACCACCCCCTGACGAACCCAAACAACCCAAACCTCCATACCTCTAGCCCTATTTACCATAGCC 5040
LWR  CCAATACAACCAACTCAACCACCCCCTGACGAACCCAAACAACCCAAACCTCCATACCTCTAGCCCTATTTACCATAGCC 5040

             5050      5060      5070      5080      5090      5100      5110      5120
LC   ACCCTTGGACTACTGCCAATGACCCCAACCATTCTAATACTAGCCACCTAGGGACTTAGGATAATATTAGACCAAAAGCC 5120
WR   ACCCTTGGACTACTACCAATGACCCCAACCATTCTAATACTAGCCACCTAGGGACTTAGGATAATATTAGACCAAAAGCC 5120
LWR  ACCCTTGGACTACTGCCAATGACCCCAACCATTCTAATACTAGCCACCTAGGGACTTAGGATAATATTAGACCAAAAGCC 5120

             5130      5140      5150      5160      5170      5180      5190      5200
LC   TTCAAAGCTTTAAGTAGAAGTGAAAATCTTCTAGTCCCTGATTAAGACCTACGAGAAATCAACTCGCATCTCCTGATTGC 5200
WR   TTCAAAGCTTTAAGTAGAAGTGAAAATCTTCTAGTCCCTGATTAAGACCTACGAGAGATCAACTCGCATCTCCTGATTGC 5200
LWR  TTCAAAGCTTTAAGTAGAAGTGAAAATCTTCTAGTCCCTGATTAAGACCTACGAGAGATCAACTCGCATCTCCTGATTGC 5200

             5210      5220      5230      5240      5250      5260      5270      5280
LC   AAATCAGATACTTTTATTAAGCTAAGGCCTTACTAGATGGGAAGGCCTCGATCCTACAAACTCTTAGTTAACAGCTAAGC 5280
WR   AAATCAGATACTTTTATTAAGCTAAGGCCTTACTAGATGGGAAGGCCTCGATCCTACAAACTCTTAGTTAACAGCTAAGC 5280
LWR  AAATCAGATACTTTTATTAAGCTAAGGCCTTACTAGATGGGAAGGCCTCGATCCTACAAACTCTTAGTTAACAGCTAAGC 5280

             5290      5300      5310      5320      5330      5340      5350      5360
LC   GCTCAAGCCAGCGAGCATCCATCTACTTTTCCCGCCGTTTAACTCAGAAAGGCGGGAAAAGCCCCGGCAGAGTATTAATC 5360
WR   GCTCAAGCCAGCGAGCATCCATCTACTTTTCCCGCCGTTTAACTCAGAAAGGCGGGAAAAGCCCCGGCAGAGTATTAATC 5360
LWR  GCTCAAGCCAGCGAGCATCCATCTACTTTTCCCGCCGTTTAACTCAGAAAGGCGGGAAAAGCCCCGGCAGAGTATTAATC 5360

             5370      5380      5390      5400      5410      5420      5430      5440
LC   TACGTCTTCGGATTTGCAATCCAATATGTTTTCTTCACCACGGGGCTGATAGGAAGAGGACTTAAACCTCTGTCTTCGGG 5440
WR   TACGTCTTCGGATTTGCAATCCAATATGTTTTCTTCACCACGGGGCTGATAGGAAGAGGACTTAAACCTCTGTCTTCGGG 5440
LWR  TACGTCTTCGGATTTGCAATCCAATATGTTTTCTTCACCACGGGGCTGATAGGAAGAGGACTTAAACCTCTGTCTTCGGG 5440

             5450      5460      5470      5480      5490      5500      5510      5520
LC   GCTACAACCCACCGCCTAAACACTCGGCTACCCTACCTGTGGCAATCACGCGCTGATTCTTCTCTACCAACCACAAAGAC 5520
WR   GCTACAACCCACCGCCTAAACACTCGGCTACCCTACCTGTGGCAATCACGCGCTGATTCTTCTCTACCAACCACAAAGAC 5520
LWR  GCTACAACCCACCGCCTAAACACTCGGCTACCCTACCTGTGGCAATCACGCGCTGATTCTTCTCTACCAACCACAAAGAC 5520

             5530      5540      5550      5560      5570      5580      5590      5600
LC   ATTGGTACCCTTTATCTAGTATTTGGTGCCTGAGCCGGAATAGTAGGAACCGCTTTAAGCCTCCTCATCCGAGCTGAACT 5600
WR   ATTGGTACCCTTTATCTAGTATTTGGTGCCTGAGCCGGAATAGTAGGAACCGCTTTAAGCCTCCTCATCCGAGCTGAACT 5600
LWR  ATTGGTACCCTTTATCTAGTATTTGGTGCCTGAGCCGGAATAGTAGGAACCGCTTTAAGCCTCCTCATCCGAGCTGAACT 5600

             5610      5620      5630      5640      5650      5660      5670      5680
LC   TAGTCAACCCGGATCACTTCTAGGTGATGACCAAATTTACAATGTAATTGTTACCGCCCACGCCTTCGTAATAATTTTCT 5680
WR   TAGTCAACCCGGATCACTTCTAGGTGATGACCAAATTTACAATGTAATTGTTACCGCCCACGCCTTCGTTATAATTTTCT 5680
LWR  TAGTCAACCCGGATCACTTCTAGGTGATGACCAAATTTACAATGTAATTGTTACCGCCCACGCCTTCGTAATAATTTTCT 5680

             5690      5700      5710      5720      5730      5740      5750      5760
LC   TTATAGTAATGCCTATCCTCATTGGAGGATTCGGAAACTGACTTGTACCCCTGATAATCGGAGCCCCAGACATGGCATTC 5760
WR   TTATAGTAATGCCTATCCTCATTGGAGGATTCGGAAACTGACTTGTGCCCCTGATAATCGGAGCCCCAGACATGGCATTC 5760
LWR  TTATAGTAATGCCTATCCTCATTGGAGGATTCGGAAACTGACTTGTACCCCTGATAATCGGAGCCCCAGACATGGCATTC 5760
```

```
          5770      5780      5790      5800      5810      5820      5830      5840
LC   CCACGAATAAATAATATAAGCTTCTGACTTCTTCCCCCATCATTCCTGTTACTACTAGCTTCCTCTGGTGTTGAAGCCGG 5840
WR   CCACGAATAAATAATATAAGCTTCTGACTTCTTCCCCCATCATTCCTGTTACTACTAGCTTCCTCTGGTGTTGAAGCCGG 5840
LWR  CCACGAATAAATAATATAAGCTTCTGACTTCTTCCCCCATCATTCCTGTTACTACTAGCTTCCTCTGGTGTTGAAGCCGG 5840

          5850      5860      5870      5880      5890      5900      5910      5920
LC   AGCTGGCACCGGATGGACAGTATACCCCCCTCTTGCAGGGAACCTGGCCCACGCAGGAGCATCAGTAGACCTAACAATTT 5920
WR   AGCTGGCACCGGATGGACAGTATACCCCCCTCTTGCAGGGAACCTAGCCCACGCAGGAGCATCAGTAGACCTAACAATTT 5920
LWR  AGCTGGCACCGGATGGACAGTATACCCCCCTCTTGCAGGAAACCTGGCCCACGCAGGAGCATCAGTAGACCTAACAATTT 5920

          5930      5940      5950      5960      5970      5980      5990      6000
LC   TCTCACTACATTTAGCAGGTGTTTCATCAATCCTGGGGGCAATCAACTTCATTACTACAACCATTAACATAAAACCTCCA 6000
WR   TCTCACTACATTTAGCAGGTGTCTCATCAATCCTGGGGGCAATCAACTTCATTACCACAACCATTAACATAAAACCTCCA 6000
LWR  TCTCACTACATTTAGCAGGTGTTTCATCAATCCTGGGGGCAATCAACTTCATTACTACAACCATTAACATAAAACCTCCA 6000

          6010      6020      6030      6040      6050      6060      6070      6080
LC   GCCATTTCCCAATACCAAACACCCCTATTTGTTTGATCCGTACTTGTAACCGCCGTCCTCCTTCTCCTATCATTACCTGT 6080
WR   GCCATTTCCCAATACCAAACACCCCTATTTGTTTGATCCGTACTTGTAACCGCCGTCCTCCTTCTCCTATCACTACCTGT 6080
LWR  GCCATTTCCCAATACCAAACACCCCTATTTGTTTGATCCGTACTTGTAACCGCCGTCCTCCTTCTCCTATCATTACCTGT 6080

          6090      6100      6110      6120      6130      6140      6150      6160
LC   TCTAGCTGCCGGTATTACAATGCTTTTAACAGATCGAAATCTTAACACCACATTCTTTGATCCCGCAGGCGGGGGAGACC 6160
WR   TCTAGCTGCCGGTATTACAATGCTTTTAACAGATCGAAATCTTAACACCACATTCTTTGACCCCGCAGGCGGGGGGGACC 6160
LWR  TCTAGCTGCCGGTATTACAATGCTTTTAACAGATCGAAATCTTAACACCACATTCTTTGATCCCGCAGGCGGGGGAGACC 6160

          6170      6180      6190      6200      6210      6220      6230      6240
LC   CAATTCTCTATCAACACTTATTCTGATTCTTTGGTCACCCAGAAGTTTACATTTTAATCCTTCCAGGATTTGGAATTATT 6240
WR   CAATTCTATATCAACACTTATTCTGATTCTTTGGTCACCCAGAAGTTTATATTTTAATCCTTCCAGGATTTGGAATTATT 6240
LWR  CAATTCTCTATCAACACTTATTCTGATTCTTTGGTCACCCAGAAGTTTATATTTTAATCCTTCCAGGATTTGGAATTATT 6240

          6250      6260      6270      6280      6290      6300      6310      6320
LC   TCTCACGTTGTAGCCTATTATTCAGGTAAAAAAGAACCATTTGGTTATATAGGAATAGTATGAGCCATAATGGCCATTGG 6320
WR   TCTCATGTTGTAGCCTATTATTCAGGTAAAAAAGAACCATTTGGTTATATAGGAATAGTATGAGCCATAATGGCCATTGG 6320
LWR  TCTCACGTTGTAGCCTATTATTCAGGTAAAAAAGAACCATTTGGTTATATAGGAATAGTATGAGCCATAATGGCCATTGG 6320

          6330      6340      6350      6360      6370      6380      6390      6400
LC   CCTCCTAGGGTTCATTGTATGAGCCCACCATATGTTTACTGTCGGAATGGACGTAGACACCCGTGCATATTTTACATCCG 6400
WR   CCTCCTAGGGTTCATTGTATGAGCCCACCATATGTTTACCGTCGGAATGGACGTAGACACCCGTGCATATTTTACATCCG 6400
LWR  CCTCCTAGGGTTCATTGTATGAGCCCACCATATGTTTACTGTCGGAATGGACGTAGACACCCGTGCATATTTTACATCCG 6400

          6410      6420      6430      6440      6450      6460      6470      6480
LC   CAACAATAATCATCGCAATTCCAACGGGTGTAAAAGTATTTAGCTGACTGGCTACACTTCACGGAGGATCAATTAAATGA 6480
WR   CAACAATAATCATCGCAATTCCAACGGGTGTAAAAGTATTTAGCTGACTGGCTACACTTCACGGAGGATCAATTAAATGA 6480
LWR  CAACAATAATCATCGCAATTCCAACGGGTGTAAAAGTATTTAGCTGACTGGCTACACTTCACGGAGGATCAATTAAATGA 6480

          6490      6500      6510      6520      6530      6540      6550      6560
LC   GAAACACCAATACTATGAGCCCTAGGATTCATTTTCCTGTTTACAGTGGGAGGACTTACAGGAATTGTCCTCTCTAATTC 6560
WR   GAAACACCAATACTATGAGCCCTAGGGTTCATCTTCCTGTTTACAGTGGGAGGACTTACAGGAATTGTCCTCTCTAATTC 6560
LWR  GAAACACCAATACTATGAGCCCTAGGATTTATTTTCCTGTTTACAGTGGGAGGACTTACAGGAATTGTCCTCTCTAATTC 6560

          6570      6580      6590      6600      6610      6620      6630      6640
LC   ATCACTTGATATTGTTCTCCACGACACCTATTATGTAGTAGCACATTTCCACTATGTACTATCAATGGGTGCCGTATTCG 6640
WR   ATCACTTGATATTGTTCTCCACGACACCTATTATGTAGTAGCACATTTCCACTATGTACTATCAATGGGTGCCGTATTCG 6640
LWR  ATCACTTGATATTGTTCTCCACGACACCTATTATGTAGTAGCACATTTCCACTATGTACTATCAATGGGTGCCGTATTCG 6640

          6650      6660      6670      6680      6690      6700      6710      6720
LC   CAATTATGGCAGCCTTTGTACACTGATTCCCCCTACTAACAGGGTACACTCTACATAGCGCTTGAACAAAAATCCACTTT 6720
WR   CAATTATGGCAGCCTTTGTACACTGATTCCCTCTACTAACAGGGTACACTCTACATAGCGCTTGGACAAAAATCCACTTT 6720
LWR  CAATTATGGCAGCCTTTGTACACTGATTCCCCCTACTAACAGGGTACACTCTACATAGCGCTTGAACAAAAATCCACTTT 6720
```

```
        6730      6740      6750      6760      6770      6780      6790      6800
LC   GGGGTTATATTTATCGGAGTTAACCTCACATTCTTCCCACAACACTTCCTGGGTCTAGCAGGAATACCACGACGGTATTC 6800
WR   GGGGTTATATTTATTGGAGTTAACCTCACATTCTTCCCACAACACTTCCTAGGTCTAGCAGGAATACCACGACGGTATTC 6800
LWR  GGGGTTATATTTATCGGAGTTAACCTCACATTCTTCCCACAACACTTCCTGGGTCTAGCAGGAATACCACGACGGTATTC 6800

        6810      6820      6830      6840      6850      6860      6870      6880
LC   TGATTATCCAGACGCTTATGCCCTATGAAATACAGTATCATCTATCGGATCCCTAATCTCCCTAGTAGCGGTAATTATGT 6880
WR   TGATTACCCAGACGCTTATGCCCTATGAAATACAGTATCATCTATCGGATCCCTAATCTCCCTAGTAGCGGTAATTATGT 6880
LWR  TGATTATCCAGACGCTTATGCCCTATGAAATACAGTATCATCTATCGGATCCCTAATCTCCCTAGTAGCGGTAATTATGT 6880

        6890      6900      6910      6920      6930      6940      6950      6960
LC   TCCTATTTATTCTATGAGAAGCCTTCGCCGCTAAACGAGAAGTATTATCTGTAGAACTAACAATAACAAATGTGGAATGA 6960
WR   TCCTATTTATCCTATGAGAAGCCTTCGCCGCTAAACGAGAAGTGTTATCTGTAGAACTAACAATAACAAATGTGGAATGA 6960
LWR  TCCTATTTATTCTATGAGAAGCCTTCGCCGCTAAACGAGAAGTGTTATCTGTAGAACTAACAATAACAAATGTGGAATGA 6960

        6970      6980      6990      7000      7010      7020      7030      7040
LC   CTCCATGGCTGCCCCCCTCCTTACCACACATACGAGGAACCAGCATTTGTTCAAATTCAATCAAATTAACGAGAAAGGGA 7040
WR   CTCCATGGCTGCCCCCCTCCTTACCACACATACGAGGAACCAGCATTTGTTCAAATTCAATCAAATTAACGAGAAAGGGA 7040
LWR  CTCCATGGCTGCCCCCCTCCTTACCACACATACGAGGAACCAGCATTTGTTCAAATTCAATCAAATTAACGAGAAAGGGA 7040

        7050      7060      7070      7080      7090      7100      7110      7120
LC   GGAATTGAACCCCCATATGCTGGTTTCAAGCCAGCCACATAACCACTCTGTCACTTCCTTCTAAAGACATTAGTAAAATG 7120
WR   GGAATTGAACCCCCATATGCTGGTTTCAAGCCAGCCACATAACCACTCTGTCACTTCCTTCTAAAGACATTAGTAAAATG 7120
LWR  GGAATTGAACCCCCATATGCTGGTTTCAAGCCAGCCACATAACCACTCTGTCACTTCCTTCTAAAGACATTAGTAAAATG 7120

        7130      7140      7150      7160      7170      7180      7190      7200
LC   AAGATTACATCACCTTGTCAAGGTGAAATTGTAGGTTAAATCCCTGCATGTCTTACACTTAAAATTAATGGCACACCCAA 7200
WR   AAGATTACATCACCTTGTCAAGGTGAAATTGTAGGTTAAATCCCTGCATGTCTTACACTTAAAATTAATGGCACACCCAA 7200
LWR  AAGATTACATCACCTTGTCAAGGTGAAATTGTAGGTTAAATCCCTGCATGTCTTACACTTAAAATTAATGGCACACCCAA 7200
```

```
        7210      7220      7230      7240      7250      7260      7270      7280
LC   CGCAACTAGGATTCCAAGACGCGGCATCACCCGTTATAGAAGAACTTCTTCACTTCCATGACCACGCATTAATAATTGTG 7280
WR   CGCAACTAGGATTCCAAGACGCGGCATCACCCGTTATAGAAGAACTTCTTCACTTCCATGACCACGCATTAATAATTGTG 7280
LWR  CGCAACTAGGATTCCAAGACGCGGCATCACCCGTTATAGAAGAACTTCTTCACTTCCATGACCACGCATTAATAATTGTG 7280

        7290      7300      7310      7320      7330      7340      7350      7360
LC   TTTTTAATTAGCACCCTAGTGCTATATATTATTATTGCAATGGTATCAACCAAACTTACTAATAAATATATTTTAGACTC 7360
WR   TTTTTAATTAGCACCCTAGTGCTATATATTATTATTGCAATGGTATCAACCAAACTTACTAATAAATATATTTTAGACTC 7360
LWR  TTTTTAATTAGCACCCTAGTGCTATATATTATTATTGCAATGGTATCAACCAAACTTACTAATAAATATATTTTAGACTC 7360

        7370      7380      7390      7400      7410      7420      7430      7440
LC   CCAAGAAATCGAAATTGTATGAACTATTCTACCAGCCGTTATTTTAGTATTAATTGCTCTACCATCCCTACGAATTCTTT 7440
WR   CCAAGAAATCGAAATTGTATGAACTATTCTACCAGCCGTTATTTTAGTATTAATTGCTCTACCATCCCTACGAATTCTTT 7440
LWR  CCAAGAAATCGAAATTGTATGAACTATTCTACCAGCCGTTATTTTAGTATTAATTGCTCTACCATCCCTACGAATTCTTT 7440

        7450      7460      7470      7480      7490      7500      7510      7520
LC   ACCTTATGGACGAAATTAATGATCCCCACCTAACAATCAAAGCAATAGGACACCAATGATACTGAAGCTATGAATATACA 7520
WR   ACCTTATGGACGAAATTAATGATCCCCACCTAACAATCAAAGCAATAGGACACCAATGATACTGAAGCTATGAGTATACA 7520
LWR  ACCTTATGGACGAAATTAATGATCCCCACCTAACAATCAAAGCAATAGGACACCAATGATACTGAAGCTATGAATATACA 7520

        7530      7540      7550      7560      7570      7580      7590      7600
LC   GATTACGAAAACCTAGGATTTGACTCTTATATAGTACCTACCCAAGACCTTGCCCCAGGACAATTCCGACTCTTAGAAAC 7600
WR   GATTACGAAAACCTAGGATTTGACTCTTATATAGTACCCACCCAAGACCTTGCCCCAGGACAATTCCGACTCTTAGAAAC 7600
LWR  GATTACGAAAACCTAGGATTTGACTCTTATATAGTACCTACCCAAGACCTTGCCCCAGGACAATTCCGACTCTTAGAAAC 7600

        7610      7620      7630      7640      7650      7660      7670      7680
LC   AGACCATCGAATAGTTGTCCCAATAGAGTCCCCAGTCCGTGTCTTAGTATCCGCTGAAGACGTACTACACTCCTGAGCCG 7680
WR   AGACCATCGAATAGTTGTCCCAATAGAGTCCCCAGTCCGTGTCTTAGTATCCGCTGAAGACGTACTACACTCCTGAGCCG 7680
LWR  AGACCATCGAATAGTTGTCCCAATAGAGTCCCCAGTCCGTGTCTTAGTATCCGCTGAAGACGTACTACACTCCTGAGCCG 7680
```

```
         7690      7700      7710      7720      7730      7740      7750      7760
LC   TTCCATCTTTAGGTGTAAAAATAGACGCAGTCCCAGGCCGACTAAATCAAACTGCTTTCATCGCCTCACGCCCAGGAGTA 7760
WR   TTCCATCTTTAGGTGTAAAAATAGACGCAGTCCCAGGCCGACTAAATCAAACTGCTTTCATCGCCTCACGCCCAGGAGTA 7760
LWR  TTCCATCTTTAGGTGTAAAAATAGACGCAGTCCCAGGCCGACTAAATCAAACTGCTTTCATCGCCTCACGCCCAGGAGTA 7760

         7770      7780      7790      7800      7810      7820      7830      7840
LC   TTCTACGGACAATGCTCTGAAATCTGCGGAGCAAATCATAGCTTTATACCAATTGTAGTTGAAGCAGTACCACTAGAACA 7840
WR   TTCTACGGACAATGCTCTGAAATCTGCGGAGCAAATCATAGCTTTATACCAATTGTAGTTGAAGCAGTACCACTAGAACA 7840
LWR  TTCTACGGACAATGCTCTGAAATCTGCGGAGCAAATCATAGCTTTATACCAATTGTAGTTGAAGCAGTACCACTAGAACA 7840

         7850      7860      7870      7880      7890      7900      7910      7920
LC   CTTCGAAAACTGATCCTCACTAATACTAGAAGACGCCTCGCTAGGAAGCTAAATATTGGACAAAGCGTTGGCCTTTTAAG 7920
WR   CTTCGAAAACTGATCCTCACTAATACTAGAAGACGCCTCGCTAGGAAGCTAAATATTGGACAAAGCGTTGGCCTTTTAAG 7920
LWR  CTTCGAAAACTGATCCTCACTAATACTAGAAGACGCCTCGCTAGGAAGCTAAATATTGGACAAAGCGTTGGCCTTTTAAG 7920

         7930      7940      7950      7960      7970      7980      7990      8000
LC   CCAAAGATTGGTGATTCCCGACCACCTCTAGTGAAATGCCACAATTAAACCCAGGCCCTTGATTTGCAATCTTAGTATTT 8000
WR   CCAAAGATTGGTGATTCCCGACCACCTCTAGTGAAATGCCACAATTAAACCCAGGCCCTTGATTTGCAATCTTAGTATTT 8000
LWR  CCAAAGATTGGTGATTCCCGACCACCTCTAGTGAAATGCCACAATTAAACCCAGGCCCTTGATTTGCAATCTTAGTATTT 8000

         8010      8020      8030      8040      8050      8060      8070      8080
LC   TCTTGACTAGTTTTCTTAACTATTATTCCAACTAAAATCTTAAGCCACATCTCACCAAATGAACCAACCCCAGTAAGTGC 8080
WR   TCTTGACTAGTTTTCTTAACTATTATTCCAACTAAAATCTTAAGCCACATCTCACCAAATGAACCAACCCCAGTAAGTGC 8080
LWR  TCTTGACTAGTTTTCTTAACTATTATTCCAACTAAAATCTTAAGCCACATCTCACCAAATGAACCAACCCCAGTAAGTGC 8080

         8090      8100      8110      8120      8130      8140      8150      8160
LC   TGAAAAACACAAAACTGAATCCTGAGACTGACCATGATAGTAAGCTTCTTCGACCAATTTGCAAGCCCATCCTACCTAGG 8160
WR   TGAAAAACACAAAACTGAATCCTGAGACTGACCATGATAGTAAGCTTCTTCGACCAATTTGCAAGCCCATCCTACCTAGG 8160
LWR  TGAAAAACACAAAACTGAATCCTGAGACTGACCATGATAGTAAGCTTCTTCGACCAATTTGCAAGCCCATCCTACCTAGG 8160
```

```
         8170      8180      8190      8200      8210      8220      8230      8240
LC   AATTCCACTAATTGCCATCGCAATTGCACTACCCTGAGTACTTTACCCAACTTCATCATCTCGATGAATTAATAACCGCC 8240
WR   AATTCCACTAATTGCCATCGCAATTGCACTGCCCTGAGTACTTTACCCAACTTCATCATCTCGATGAATTAATAACCGCC 8240
LWR  AATTCCACTAATTGCCATCGCAATTGCACTACCCTGAGTACTTTACCCAACTTCATCATCTCGATGAATTAATAACCGCC 8240

         8250      8260      8270      8280      8290      8300      8310      8320
LC   TTATTACAATTCAAGGATGATTTATTAATCGATTTACAAATCAACTAATACTCCCACTAAATGTAGGAGGACATAAATGA 8320
WR   TTATTACAATTCAAGGATGATTTATTAATCGATTTACAAATCAGCTAATACTCCCACTAAATGTAGGGGGACATAAATGA 8320
LWR  TTATTACAATTCAAGGATGATTTATTAATCGATTTACAAATCAACTAATACTCCCACTAAATGTAGGAGGACATAAATGA 8320

         8330      8340      8350      8360      8370      8380      8390      8400
LC   GCCCTACTACTAGCCTCCCTAATAATTTTCTTAATTACAATTAACATGTTAGGGCTCCTGCCATATACCTTTACACCAAC 8400
WR   GCCCTACTACTAGCCTCCCTAATAATTTTCTTAATTACAATTAACATGTTAGGGCTCCTGCCATATACCTTTACACCAAC 8400
LWR  GCCCTACTACTAGCCTCCCTAATAATTTTCTTAATTACAATTAACATGTTAGGGCTCCTGCCATATACCTTTACACCAAC 8400

         8410      8420      8430      8440      8450      8460      8470      8480
LC   GACACAACTATCACTCAATATAGGATTTGCTGTACCACTATGACTTGCTACAGTAATTATCGGAATACGAAATCAACCAA 8480
WR   GACACAACTATCACTCAATATAGGATTTGCTGTACCACTATGACTTGCTACAGTAATTATCGGAATGCGAAATCAACCAA 8480
LWR  GACACAACTATCACTCAATATAGGATTTGCTGTACCACTATGACTTGCTACAGTAATTATCGGAATACGAAATCAACCAA 8480

         8490      8500      8510      8520      8530      8540      8550      8560
LC   CAGTAGCTTTAGGTCACCTGTTACCAGAAGGTACACCCATCCCACTGATTCCAGTACTAATTATTATCGAAACAATTAGT 8560
WR   CAGTAGCTTTAGGTCACCTGTTACCAGAAGGTACACCCATCCCACTGATTCCAGTACTAATTATTATCGAAACAATTAGT 8560
LWR  CAGTAGCTTTAGGTCACCTGTTACCAGAAGGTACACCCATCCCACTGATTCCAGTACTAATTATTATCGAAACAATTAGT 8560

         8570      8580      8590      8600      8610      8620      8630      8640
LC   CTATTTATTCGACCATTAGCCCTAGGAGTCCGACTCACAGCCAACTTAACTGCAGGCCACCTTCTAATTCAACTTATTGC 8640
WR   CTATTTATTCGACCATTAGCCCTAGGAGTCCGACTCACAGCCAACTTAACTGCAGGCCACCTTCTAATTCAACTTATCGC 8640
LWR  CTATTTATTCGACCATTAGCCCTAGGAGTCCGACTCACAGCCAACTTAACTGCAGGCCACCTTCTAATTCAACTTATTGC 8640
```

```
        8650      8660      8670      8680      8690      8700      8710      8720
LC   CACAGCTGTATTTGTTCTCCTACCAATGATACCAACAGTAGCAATTCTAACCGCCACAGTATTATTTTTACTAACACTAT 8720
WR   CACAGCTGTATTTGTCCTACTACCAATGATACCAACAGTAGCAATTCTAACCGCCGCAGTATTATTTTTACTAACACTAT 8720
LWR  CACAGCTGTATTTGTTCTCCTACCAATGATACCAACAGTAGCAATTCTAACCGCCACAGTATTATTTTTACTAACACTAT 8720

        8730      8740      8750      8760      8770      8780      8790      8800
LC   TAGAAGTAGCCGTAGCAATAATTCAAGCTTATGTATTTGTACTTCTTCTAAGCCTATACCTACAAGAAAACGTTTAATGG 8800
WR   TAGAAGTAGCCGTGGCAATAATTCAAGCTTATGTATTTGTACTTCTTCTAAGCCTATACCTACAAGAAAACGTTTAATGG 8800
LWR  TAGAAGTAGCCGTAGCAATAATTCAAGCTTATGTATTTGTACTTCTTCTAAGCCTATACCTACAAGAAAACGTTTAATGG 8800

        8810      8820      8830      8840      8850      8860      8870      8880
LC   CCCACCAAGCACATGCCTATCATATAGTTGATCCAAGCCCATGACCACTAACCGGAGCTATCGCTGCCCTACTAATAACA 8880
WR   CCCACCAAGCACATGCCTATCATATAGTTGATCCAAGCCCATGACCACTAACCGGAGCTATCGCTGCCCTACTAATAACA 8880
LWR  CCCACCAAGCACATGCCTATCATATAGTTGATCCAAGCCCATGACCACTAACCGGAGCTATCGCTGCCCTACTAACAACA 8880

        8890      8900      8910      8920      8930      8940      8950      8960
LC   TCCGGCTTAGCAATCTGATTTCACTTCCACTCAACAACATTAATAACTCTAGGACTAATTCTCCTACTTCTTACTATGTA 8960
WR   TCCGGCTTAGCAATCTGATTTCACTTCCACTCAACAACATTAATAACTCTAGGACTAATTCTCCTACTTCTTACTATGTA 8960
LWR  TCCGGCTTAGCAATCTGATTTCACTTCCACTCAACAACATTAATAACTCTAGGACTAATTCTCCTACTTCTTACTATGTA 8960

        8970      8980      8990      9000      9010      9020      9030      9040
LC   TCAATGATGACGTGACATCATTCGAGAAGGAACCTTCCAAGGCCACCACACACCCCCAGTACAAAAAGGACTACGATACG 9040
WR   TCAATGATGACGTGACATCATTCGAGAAGGAACCTTCCAAGGCCACCACACACCCCCAGTACAAAAAGGACTACGATACG 9040
LWR  TCAATGATGACGTGACATCATTCGAGAAGGAACCTTCCAAGGCCACCACACACCCCCAGTACAAAAAGGACTACGATACG 9040

        9050      9060      9070      9080      9090      9100      9110      9120
LC   GAATAATCTTATTTATTACCTCCGAAGTATTCTTCTTCCTAGGATTCTTCTGAGCATTCTACCACTCGAGTTTAGCACCT 9120
WR   GAATAATCTTATTTATTACCTCCGAAGTATTCTTCTTCCTAGGATTCTTCTGAGCATTCTACCACTCAAGTTTAGCACCT 9120
LWR  GAATAATCTTATTTATTACCTCCGAAGTATTCTTCTTCCTAGGATTCTTCTGAGCATTCTACCACTCGAGTTTAGCACCT 9120
```

```
        9130      9140      9150      9160      9170      9180      9190      9200
LC   ACCCCAGAACTAGGTGGATGCTGACCTCCCACAGGAATCACCCCCCTAGACCCATTTGAAGTGCCCCTTCTCAACACAGC 9200
WR   ACCCCAGAACTAGGGGGATGCTGACCTCCCACAGGAATCACCCCCCTAGACCCATTTGAAGTGCCCCTTCTCAACACAGC 9200
LWR  ACCCCAGAACTAGGTGGATGCTGACCTCCCACAGGAATCACCCCCCTAGACCCATTTGAAGTGCCCCTTCTCAACACAGC 9200

        9210      9220      9230      9240      9250      9260      9270      9280
LC   TGTACTATTAGCATCCGGGGTTACAGTTACATGAGCCCACCACAGCATTATAGAAGGGGAACGAAAACAAGCTATTCAAT 9280
WR   TGTACTATTAGCATCAGGGGTTACAGTTACATGAGCCCACCACAGCATTATAGAAGGGGAACGAAAACAAGCTATTCAAT 9280
LWR  TGTACTATTAGCATCCGGGGTTACAGTTACATGAGCCCACCACAGCATTATAGAAGGGGAACGAAAACAAGCTATTCAAT 9280

        9290      9300      9310      9320      9330      9340      9350      9360
LC   CCCTAGCATTAACCATTCTACTTGGACTCTACTTTACTGCTCTCCAAGCTATGGAATATTATGAAGCACCCTTCACAATT 9360
WR   CCTTAGCATTAACCATTCTACTTGGACTCTACTTTACTGCTCTCCAAGCTATGGAATATTATGAAGCACCATTCACAATT 9360
LWR  CCCTAGCATTAACCATTCTACTTGGACTCTACTTTACTGCTCTCCAAGCTATGGAATATTATGAAGCACCCTTCACAATT 9360

        9370      9380      9390      9400      9410      9420      9430      9440
LC   GCAGACGGAGTATATGGCTCAACATTCTTTGTAGCTACAGGATTCCATGGACTCCATGTTATTATTGGATCAACCTTCCT 9440
WR   GCAGACGGAGTATATGGCTCAACATTCTTTGTAGCTACAGGGTTCCATGGACTCCATGTTATTATTGGATCAACCTTCCT 9440
LWR  GCAGACGGAGTATATGGCTCAACATTCTTTGTAGCTACAGGATTCCATGGACTCCATGTTATTATTGGATCAACCTTCCT 9440

        9450      9460      9470      9480      9490      9500      9510      9520
LC   AGCAGTATGCCTTCTACGCCAAATCCAATACCACTTTACATCCGAACACCATTTCGGCTTTGAAGCCGCTGCCTGATACT 9520
WR   AGCAGTATGCCTTCTACGCCAAATCCAATACCACTTTACATCCGAACACCATTTCGGCTTTGAAGCCGCTGCCTGATACT 9520
LWR  AGCAGTATGCCTTCTACGCCAAATCCAATACCACTTTACATCCGAACACCATTTCGGCTTTGAAGCCGCTGCCTGATACT 9520

        9530      9540      9550      9560      9570      9580      9590      9600
LC   GACACTTTGTCGACGTAGTATGACTATTCCTCTACGTGTCTATCTATTGATGAGGCTCATAATCTTTCTAGTATTAAAGT 9600
WR   GACACTTTGTCGACGTAGTATGACTATTCCTCTACGTGTCTATCTATTGATGAGGCTCATAATCTTTCTAGTATTAAAGT 9600
LWR  GACACTTTGTCGACGTAGTATGACTATTCCTCTACGTGTCTATCTATTGATGAGGCTCATAATCTTTCTAGTATTAAAGT 9600
```

```
     9610 9620 9630 9640 9650 9660 9670 9680
LC   TAGTACAAGTGACTTCCAATCACACAGTCTTGGTTAAACCCCAAGGAAAGATAATGAATCTAATTATAACCATTTTAACT 9680
WR   TAGTACAAGTGACTTCCAATCACACAGTCTTGGTTAAACCCCAAGGAAAGATAATGAATCTAATTATAACCATTTTAACT 9680
LWR  TAGTACAAGTGACTTCCAATCACACAGTCTTGGTTAAACCCCAAGGAAAGATAATGAATCTAATTATAACTATTTTAACT 9680

     9690 9700 9710 9720 9730 9740 9750 9760
LC   ATTACAGCCGCCCTTTCACTAATTTTAGCAACCATTTCTTTCTGACTCCCACAAATAAACCCAGACGCAGAAAAGCTATC 9760
WR   ATTACAGCCGCCCTTTCACTAATTTTAGCAACCATTTCTTTCTGACTCCCACAAATAAACCCAGACGCAGAAAAGCTATC 9760
LWR  ATTACAGCCGCCCTTTCACTAATTTTAGCAACCATTTCTTTCTGACTCCCACAAATAAACCCAGACGCAGAAAAGCTATC 9760

     9770 9780 9790 9800 9810 9820 9830 9840
LC   ACCATACGAATGTGGATTTGACCCACTGGGATCTGCCCGATTGCCATTTTCTTTACGCTTCTTCCTAGTAGCTATTTTAT 9840
WR   ACCATACGAATGTGGATTTGACCCCCTGGGATCTGCCCGATTGCCATTTTCTTTACGCTTCTTCCTAGTAGCTATTTTAT 9840
LWR  ACCATACGAATGTGGATTTGATCCACTGGGATCTGCCCGATTGCCATTTTCTTTACGCTTCTTCCTAGTAGCTATTTTAT 9840

     9850 9860 9870 9880 9890 9900 9910 9920
LC   TTCTTCTCTTTGACCTAGAAATTGCCCTTCTCCTCCCATTACCCTGAGGGGATCAACTTAACAATCCTACTGGAACATTC 9920
WR   TTCTTCTCTTTGACCTAGAAATTGCCCTTCTCCTCCCATTACCCTGAGGGGACCAACTTAACAATCCTACTGGAACATTC 9920
LWR  TTCTTCTCTTTGACCTAGAAATTGCCCTTCTCCTCCCATTACCCTGAGGGGATCAACTTAACAATCCTACTGGAACATTC 9920

     9930 9940 9950 9960 9970 9980 9990 10000
LC   TTCTGAGCCACAACAGTCCTAATCTTATTAACCCTAGGACTAATTTATGAATGAACTCAAGGTGGCTTAGAATGAGCAGA 10,000
WR   TTCTGAGCCACAACAGTCCTAATCTTATTAACCCTGGGACTAATTTATGAATGAACTCAAGGTGGCTTAGAATGAGCAGA 10,000
LWR  TTCTGAGCCACAACAGTCCTAATCTTATTAACCCTAGGACTAATTTATGAATGAACTCAAGGTGGCTTAGAATGAGCAGA 10,000

     10010 10020 10030 10040 10050 10060 10070 10080
LC   ATAGGGAGCTAGTCCAAAACAAGACCTCTGATTTCGGCTCAGAAAATCGTGGTTTAATTCCACGGCCCCCTTATGACACC 10,080
WR   ATAGGGAGCTAGTCCAAAACAAGACCTCTGATTTCGGCTCAGAAAATCGTGGTTTAATTCCACGGCCCCCTTATGACACC 10,080
LWR  ATAGGGAGCTAGTCCAAAACAAGACCTCTGATTTCGGCTCAGAAAATCGTGGTTTAATTCCACGGCCCCCTTATGACACC 10,080

     10090 10100 10110 10120 10130 10140 10150 10160
LC   CGTACATTTTAGCTTTAGCTCAGCATTCATCTTAGGCCTAATAGGACTGGCATTTCACCGGACCCACCTACTCTCCGCAC 10,160
WR   CGTACATTTTAGCTTTAGCTCAGCATTCATCTTAGGCCTGATAGGACTGGCATTTCACCGGACCCACCTACTCTCCGCAC 10,160
LWR  CGTACATTTTAGCTTTAGCTCAGCATTCATCTTAGGCCTAATAGGACTGGCATTTCACCGGACCCACCTACTCTCCGCAC 10,160

     10170 10180 10190 10200 10210 10220 10230 10240
LC   TCTTATGTTTAGAAGGAATAATATTATCCCTATTTATTGCACTGGCCCTATGAGCCCTACAATTTGAATCTACAGGATTT 10,240
WR   TCTTATGTTTAGAAGGAATAATATTATCCCTATTTATTGCACTGGCCCTATGAGCCCTACAATTTGAATCTACAGGATTT 10,240
LWR  TCTTATGTTTAGAAGGAATAATATTATCCCTATTTATTGCACTGGCCCTATGAGCCCTACAATTTGAATCTACAGGATTT 10,240

     10250 10260 10270 10280 10290 10300 10310 10320
LC   TCAACGGCCCCCATATTACTTTTGGCCTTCTCTGCTTGCGAAGCTAGCACTGGTCTGGCCCTACTAGTTGCCACTGCTCG 10,320
WR   TCAACAGCCCCCATATTACTTTTGGCCTTCTCTGCTTGCGAAGCTAGCACTGGTCTGGCCTTACTAGTTGCCACTGCTCG 10,320
LWR  TCAACGGCCCCCATATTACTTTTGGCCTTCTCTGCTTGCGAAGCTAGCACTGGTCTGGCCCTACTAGTTGCCACTGCTCG 10,320

     10330 10340 10350 10360 10370 10380 10390 10400
LC   TACCCACGGCACTGACCGACTACAAAACCTCAATCTTCTACAATGCTAAAAGTACTTATTCCCACATTTATATTATTCCC 10,400
WR   TACCCACGGCACTGACCGACTACAAAACCTCAATCTTCTACAATGCTAAAAGTACTTATTCCCACATTTATACTATTCCC 10,400
LWR  TACCCACGGCACTGACCGACTACAAAACCTCAATCTTCTACAATGCTAAAAGTACTTATTCCCACATTTATATTATTCCC 10,400

     10410 10420 10430 10440 10450 10460 10470 10480
LC   AACAATTTGATTAACTTCTCCTAAATGACTATGGACAGCTACAACCGCACACAGCCTCCTAATTGCCTCCATTAGCCTAA 10,480
WR   AACAATTTGATTAACTTCTCCTAAATGACTATGGACAACTACAACCGCACACAGCCTCCTAATTGCCTCCATTAGCCTAA 10,480
LWR  AACAATTTGATTAACTTCTCCTAAATGACTATGGACAGCTACAACCGCACACAGCCTCCTAATTGCCTCCATTAGCCTAA 10,480

     10490 10500 10510 10520 10530 10540 10550 10560
LC   TATGATTTAAATGGACATCTGAAACTGGATGAACTTCCTCCAACACATACTTAGCCACAGATCCACTATCAACCCCCCTT 10,560
WR   TATGATTTAAATGGACATCTGAAACCGGATGAACTTCCTCCAACACATACTTAGCCACAGATCCACTATCAACCCCCCTT 10,560
LWR  TATGATTTAAATGGACATCTGAAACTGGATGAACTTCCTCCAACACATACTTAGCCACAGATCCACTATCAACCCCCCTT 10,560
```

```
        10570     10580     10590     10600     10610     10620     10630     10640
LC  TTAGTACTAACATGCTGACTACTTCCCCTTATAATTTTAGCCAGCCAAAACCACATTAATCCAGAGCCAATTAGCCGACA 10,640
WR  TTAGTACTAACATGCTGACTACTTCCCCTTATAATTTTAGCCAGCCAAAACCACATTAATCCAGAGCCAATTAGCCGACA 10,640
LWR TTAGTACTAACATGCTGACTACTTCCCCTTATAATTTTAGCCAGCCAAAACCACATTAATCCAGAGCCAATTAGCCGACA 10,640

        10650     10660     10670     10680     10690     10700     10710     10720
LC  ACGATTGTATATTACGCTCCTGGCCTCACTACAAGCCTTTCTAATTATAGCATTCGGCGCCACAGAAATTATTATATTTT 10,720
WR  ACGACTGTATATTACGCTCCTGACCTCACTACAAGCCTTTCTAATTATAGCATTCGGCGCCACAGAAATTATTATATTTT 10,720
LWR ACGATTGTATATTACGCTCCTGGCCTCACTACAAGCCTTTCTAATTATAGCATTCGGCGCCACAGAAATTATTATATTTT 10,720

        10730     10740     10750     10760     10770     10780     10790     10800
LC  ATATTATATTTGAAGCTACACTTATCCCAACCCTTATCATTATTACCCGATGAGGAAATCAAACCGAACGACTCAATGCA 10,800
WR  ATATTATATTTGAAGCTACACTTATCCCAACCCTTATCATCATTACCCGATGAGGAAATCAGACCGAACGACTCAATGCA 10,800
LWR ATATTATATTTGAAGCTACACTTATCCCAACCCTTATCATTATTACCCGATGAGGAAATCAAACCGAACGACTCAATGCA 10,800

        10810     10820     10830     10840     10850     10860     10870     10880
LC  GGAACCTACTTCTTGTTTTATACCCTAGCAGGATCACTCCCGCTCTTAGTTGCCCTTCTCCTTCTCCAGCAATCTACGGG 10,880
WR  GGAACCTACTTCTTATTTTATACCCTAGCAGGATCACTCCCGCTCTTAGTTGCCCTTCTCCTTCTCCAGCAATCTACGGG 10,880
LWR GGAACCTACTTCTTGTTTTATACCCTAGCAGGATCACTCCCGCTCTTAGTTGCCCTTCTCCTTCTCCAGCAATCTACGGG 10,880

        10890     10900     10910     10920     10930     10940     10950     10960
LC  CACACTATCAATATTAGTTCTCCAATATTCACAACCTCTACAACTCAATTCTTGAGGCCATATAATCTGATGAGCTGGCT 10,960
WR  CACACTATCAATACTAGTTCTCCAATATTCACAACCTCTACAACTCAATTCTTGAGGCCATATAATCTGATGAGCTGGCT 10,960
LWR CACACTATCAATATTAGTTCTCCAATATTCACAACCTCTACAACTCAATTCTTGAGGCCATATAATCTGATGAGCTGGCT 10,960

        10970     10980     10990     11000     11010     11020     11030     11040
LC  GCCTAATCGCATTTTTAGTTAAAATACCATTATATGGAGTACACCTTTGACTACCAAAAGCACATGTAGAAGCCCCTGTA 11,040
WR  GCCTAATCGCATTTTTAGTTAAAATACCATTATATGGAGTACACCTTTGACTACCAAAAGCACATGTAGAAGCCCCTGTA 11,040
LWR GCCTAATCGCATTTTTAGTTAAAATACCATTATATGGAGTACACCTTTGACTACCAAAAGCACATGTAGAAGCCCCTGTA 11,040
```

```
        11050     11060     11070     11080     11090     11100     11110     11120
LC  GCAGGATCAATAGTACTAGCAGCAGTCTTACTAAAGCTAGGTGGATACGGAATAATACGAATAATAGTGATATTAGACCC 11,120
WR  GCAGGATCAATAGTATTAGCAGCAGTCTTACTAAAACTAGGTGGATACGGAATAATACGAATAATAGTGATATTAGACCC 11,120
LWR GCAGGATCAATAGTACTAGCAGCAGTCTTACTAAAGCTAGGTGGATACGGAATAATACGAATAATAGTGATATTAGACCC 11,120

        11130     11140     11150     11160     11170     11180     11190     11200
LC  CCTATCAAAAGAACTGGCCTACCCATTTATTATTTTAGCCCTATGAGGCATTATTATAACAGGGTCAATTTGCCTCCGAC 11,200
WR  CCTATCAAAAGAACTAGCCTACCCATTTATTATTTTAGCCCTATGAGGCATTATTATAACAGGGTCAATTTGCCTCCGAC 11,200
LWR CCTATCAAAAGAACTGGCCTACCCATTTATTATTTTAGCCCTATGAGGCATTATTATAACAGGGTCAATTTGCCTCCGAC 11,200

        11210     11220     11230     11240     11250     11260     11270     11280
LC  AAACAGACCTAAAATCACTAATCGCCTACTCATCCGTAAGTCACATGGGCCTTGTAGCAGGCGGAATTTTAATCCAAACC 11,280
WR  AAACAGACCTAAAATCACTAATCGCCTACTCATCCGTAAGTCACATAGGCCTTGTAGCAGGCGGGATTTTAATCCAAACC 11,280
LWR AAACAGACCTAAAATCACTAATCGCCTACTCATCCGTAAGTCACATGGGCCTTGTAGCAGGCGGAATTTTAATCCAAACC 11,280

        11290     11300     11310     11320     11330     11340     11350     11360
LC  CCATGAGGATTTTCAGGAGCAATTATTTTAATAATTGCCCACGGATTAGTCTCCTCAGCACTATTTTGCTTAGCCAACAC 11,360
WR  CCATGAGGATTTTCAGGAGCAATTATTTTAATAATTGCCCATGGATTAGTCTCCTCAGCACTATTTTGCTTAGCCAACAC 11,360
LWR CCATGAGGATTTTCAGGAGCAATTATTTTAATAATTGCCCACGGATTAGTCTCCTCAGCACTATTTTGCTTAGCCAACAC 11,360

        11370     11380     11390     11400     11410     11420     11430     11440
LC  AGCTTATGAACGAACACACAGCCGAACAATAATCCTTGCCCGAGGACTACAAATTATTTTTCCACTAACCGCAGTATGGT 11,440
WR  AGCTTATGAACGAACACACAGCCGAACAATAATCCTTGCCCGAGGACTACAAATTATTTTTCCACTAACCGCAGTATGGT 11,440
LWR AGCTTATGAACGAACACACAGCCGAACAATAATCCTTGCCCGAGGACTACAAATTATTTTTCCACTAACCGCAGTATGGT 11,440

        11450     11460     11470     11480     11490     11500     11510     11520
LC  GATTCATTGCTAACCTAGCTAACCTAGCACTCCCACCACTGCCAAACCTAATAGGAGAACTTATAATCATTACAACACTA 11,520
WR  GATTCATTGCTAACCTAGCTAACCTAGCACTCCCACCACTACCCAACCTAATAGGAGAACTTATAATCATTACAACACTA 11,520
LWR GATTCATTGCTAACCTAGCTAACCTAGCACTCCCACCACTGCCAAACCTAATAGGAGAACTTATAATCATTACAACACTA 11,520
```

```
          11530     11540     11550     11560     11570     11580     11590     11600
LC   TTTAACTGATCCCCATGAACAATTCTACTTACAGGTCTAGGAACACTTATTACAGCTGGCTACTCCCTATATATGTTCCT 11,600
WR   TTTAACTGATCCCCATGAACGATTCTACTTACAGGTCTAGGAACACTTATTACAGCTGGCTACTCCCTATATATGTTCCT 11,600
LWR  TTTAACTGATCCCCATGAACAATTCTACTTACAGGTCTAGGAACACTTATTACAGCTGGCTACTCCCTATATATGTTCCT 11,600

          11610     11620     11630     11640     11650     11660     11670     11680
LC   TATATCACAACGAGGCCCTACACCAAATCACATTACGGGACTCCAACCATTTCATACCCGAGAACACCTACTGATAACAC 11,680
WR   TATATCACAACGAGGCCCTACACCAAATCACATTACAGGACTCCAACCATTTCATACCCGAGAACACCTACTGATAATAC 11,680
LWR  TATATCACAACGAGGCCCTACACCAAATCACATTACGGGACTCCAACCATTTCATACCCGAGAACACCTACTGATAACAC 11,680

          11690     11700     11710     11720     11730     11740     11750     11760
LC   TACACCTAGTTCCCGTCATCCTATTGGTAACAAAACCAGAACTCATATGAGGATGATGCTACTGTAAGTATAGTTTAGCC 11,760
WR   TACACCTAGTTCCCGTCATCCTATTGGTAACAAAACCAGAACTCATATGAGGATGATGCTACTGTAAGTATAGTTTAACC 11,760
LWR  TACACCTAGTTCCCGTCATCCTATTGGTAACAAAACCAGAACTCATATGAGGATGATGCTACTGTAAGTATAGTTTAGCC 11,760

          11770     11780     11790     11800     11810     11820     11830     11840
LC   AAAATATTAGATTGTGATTCTAAAGATAGGAGTTAAAGTCTCCTTACTCACCAAGGGAGAACAGAAAATAGCAAGCACTG 11,840
WR   AAAATATTAGATTGTGATTCTAAAGATAGGAGTTAAAATCTCCTTACTCACCAAGGAAGAACAGAAAATAGCAAGCACTG 11,840
LWR  AAAATATTAGATTGTGATTCTAAAGATAGGAGTTAAAGTCTCCTTACTCACCAAGGGAGAACAGAAAATAGCAAGCACTG 11,840

          11850     11860     11870     11880     11890     11900     11910     11920
LC   CTAATCCTTGCCCTCCATGGTTAAAATCCATGGCTTCCTTGCGCTTTTAAAGGATAACAGTTCATCCGTTGGTCTTAGGA 11,920
WR   CTAATCCTTGCCCCCCATGGTTAAAATCCGTGGCTTCCTTGCGCTTTTAAAGGATAACAGTTCATCCGTTGGTCTTAGGA 11,920
LWR  CTAATCCTTGCCCTCCATGGTTAAAATCCATGGCTTCCTTGCGCTTTTAAAGGATAACAGTTCATCCGTTGGTCTTAGGA 11,920

          11930     11940     11950     11960     11970     11980     11990     12000
LC   ACCAAAAACTCTTGGTGCAAATCCAAGTAGAAGCTAAAATGACATTAATTATACACTCATCACTTCTCCTAATCTTTTTT 12,000
WR   ACCAAAAACTCTTGGTGCAAATCCAAGTAGAAGCTAAAATGACATTAATTATACACTCATCACTTCTCCTAATCTTTTTT 12,000
LWR  ACCAAAAACTCTTGGTGCAAATCCAAGTAGAAGCTAAAATGACATTAATTATACACTCATCACTTCTCCTAATCTTTTTT 12,000
```

```
          12010     12020     12030     12040     12050     12060     12070     12080
LC   ATTTTAGTGTACCCGCTACTTACTACATTAAACCCCAACCAACAAGGATCAAACATAGCAGGAATAACCAAAATTGCCGT 12,080
WR   ATTTTAGTGTACCCGCTACTTACTACATTAAACCCCAACCAACAAGGGTCAAACATAGCAGGAATAACCAAAGTTGCCGT 12,080
LWR  ATTTTAGTGTACCCGCTACTTACTACATTAAACCCCAACCAACAAGGATCAAACATAGCAGGAATAACCAAAATTGCCGT 12,080

          12090     12100     12110     12120     12130     12140     12150     12160
LC   TAGTTCCGCATTCTTCATTAGTCTCTTACCATTAATAATTTTTCTGAATTTAAAAACAGAAGGAATTATCACAAACTGAC 12,160
WR   TAGTTCCGCATTCTTCATTAGTCTCTTACCATTAATAATTTTTCTGAATTTAAAAACAGAAGGAATTATTACAAACTGAC 12,160
LWR  TAGTTCCGCATTCTTCATTAGTCTCTTACCATTAATAATTTTTCTGAATTTAAAAACAGAAGGAATTATCACAAACTGAC 12,160

          12170     12180     12190     12200     12210     12220     12230     12240
LC   AATGAATGAATACCCAAACATTTGACGTAAACATCAGCTTTAAATTTGACCACTACTCCCTAATTTTTGTCCCAATTGCC 12,240
WR   AATGAATAAATACCCAAACATTTGACGTAAACATCAGCTTTAAATTTGACCACTACTCCCTAATTTTTGTCCCAATTGCC 12,240
LWR  AATGAATGAATACCCAAACATTTGACGTAAACATCAGCTTTAAATTTGACCACTACTCCCTAATTTTTGTCCCAATTGCC 12,240

          12250     12260     12270     12280     12290     12300     12310     12320
LC   CTCTATGTAACCTGATCAATTCTAGAGTTCGCACTGTGATATATACACTCCGACCCCTATATCGACCGATTCTTTAAATA 12,320
WR   CTCTATGTAACCTGATCAATTCTAGAGTTCGCACTGTGATATATACACTCCGACCCCTATATTGACCGATTCTTTAAATA 12,320
LWR  CTCTATGTAACCTGATCAATTCTAGAGTTCGCACTGTGATATATACACTCCGACCCCTATATCGACCGATTCTTTAAATA 12,320

          12330     12340     12350     12360     12370     12380     12390     12400
LC   TCTACTCACGTTTTTAGTAGCCATAATTATTCTAGTTACAGCTAACAATATATTTCAACTATTTATTGGTTGAGAAGGAG 12,400
WR   TCTACTCACGTTTTTAGTAGCCATAATTATTCTAGTTACAGCTAACAATATATTTCAACTATTTATTGGTTGAGAAGGAG 12,400
LWR  TCTACTCACGTTTTTAGTAGCCATAATTATTCTAGTTACAGCTAACAATATATTTCAACTATTTATTGGTTGAGAAGGAG 12,400

          12410     12420     12430     12440     12450     12460     12470     12480
LC   TAGGAATCATATCCTTCTTACTTATCGGATGATGGCACGGACGGGCAGATGCCAACACAGCCGCTCTTCAAGCTGTAATC 12,480
WR   TAGGAATCATATCCTTCTTACTTATCGGATGATGACACGGACGGGCAGATGCCAACACAGCCGCTCTTCAAGCTGTAATC 12,480
LWR  TAGGAATCATATCCTTCTTACTTATCGGATGATGGCACGGACGGGCAGATGCCAACACAGCCGCTCTTCAAGCTGTAATC 12,480
```

```
                 12490     12500     12510     12520     12530     12540     12550     12560
LC    TACAACCGGGTAGGAGACATCGGGTTAATCATAACTATGGCCTGATTTGCAATGAACCTTAACTCCTGAGAAATTCAACA 12,560
WR    TACAACCGAGTAGGGGACATCGGGTTAATCATAACTATGGCCTGATTTGCAATAAACCTTAACTCCTGAGAAATTCAACA 12,560
LWR   TACAACCGGGTAGGAGACATCGGGTTAATCATAACTATGGCCTGATTTGCAATGAACCTTAACTCCTGAGAAATTCAACA 12,560

                 12570     12580     12590     12600     12610     12620     12630     12640
LC    AATCTTTGTCTTATCAAAAAACTTCGATTTAACAATTCCCTTAATAGGACTTGCTCTAGCAGCAACGGGAAAATCAGCCC 12,640
WR    AATCTTTGTCTTATCAAAAAACTTCGATTTAACAATTCCCTTAATAGGACTTGCTCTAGCAGCGACGGGAAAATCAGCCC 12,640
LWR   AATCTTTGTCTTATCAAAAAACTTCGATTTAACAATTCCCTTAATAGGACTTGCTCTAGCAGCAACGGGAAAATCAGCCC 12,640

                 12650     12660     12670     12680     12690     12700     12710     12720
LC    AATTTGGCCTCCACCCCTGACTTCCCTCCGCCATGGAGGGCCCTACACCAGTATCTGCCCTACTCCACTCAAGTACTATA 12,720
WR    AATTTGGCCTCCACCCCTGACTTCCCTCCGCCATGGAGGGCCCTACACCAGTATCTGCCCTACTCCACTCAAGTACCATA 12,720
LWR   AATTTGGCCTCCACCCCTGACTTCCCTCCGCCATGGAGGGCCCTACACCAGTATCTGCCCTACTCCACTCAAGTACTATA 12,720

                 12730     12740     12750     12760     12770     12780     12790     12800
LC    GTTGTTGCAGGAATTTTCCTGCTTATCCGCCTTCACCCCCTCATAGAAAATAACCAGTTAGCTCTAACAACCTGCCTATG 12,800
WR    GTTGTTGCAGGAATTTTCCTACTTATCCGCCTTCACCCCCTCATAGAAAATAACCAGTTAGCTCTAACAACCTGCCTATG 12,800
LWR   GTTGTTGCAGGAATTTTCCTGCTTATCCGCCTTCACCCCCTCATAGAAAATAACCAGTTAGCTCTAACAACCTGCCTATG 12,800

                 12810     12820     12830     12840     12850     12860     12870     12880
LC    CCTTGGAGCACTAACCTCACTATTCACAGCCACCTGTGCTCTAACCCAAAACGATATCAAGAAAATTGTAGCTTTCTCAA 12,880
WR    CCTTGGAGCACTAACCTCACTATTCACAGCCACCTGTGCTCTGACCCAAAACGATATCAAGAAAATTGTAGCTTTCTCAA 12,880
LWR   CCTTGGAGCACTAACCTCACTATTCACAGCCACCTGTGCTCTAACCCAAAACGATATCAAGAAAATTGTAGCTTTCTCAA 12,880

                 12890     12900     12910     12920     12930     12940     12950     12960
LC    CATCCAGTCAATTAGGCCTAATAATAGTTACAATTGGACTAAACCAACCACAACTAGCATTCCTTCACATCTGCACCCAT 12,960
WR    CATCTAGTCAATTAGGCCTAATAATAGTTACAATTGGACTAAACCAACCACAACTAGCATTCCTTCACATCTGCACCCAT 12,960
LWR   CATCCAGTCAATTAGGCCTAATAATAGTTACAATTGGACTAAACCAACCACAACTAGCATTCCTTCACATCTGCACCCAT 12,960
```

```
                 12970     12980     12990     13000     13010     13020     13030     13040
LC    GCTTTCTTTAAAGCCATACTATTCCTATGCTCAGGGTCCATTATTCACAGCCTAAACGACGAACAAGACATCCGAAAAAT 13,040
WR    GCTTTCTTTAAAGCCATACTATTCCTATGCTCAGGGTCCATTATTCACAGCCTAAACGACGAACAAGATATCCGAAAAAT 13,040
LWR   GCTTTCTTTAAAGCCATACTATTCCTATGCTCAGGGTCCATTATTCACAGCCTAAACGACGAACAAGACATCCGAAAAAT 13,040

                 13050     13060     13070     13080     13090     13100     13110     13120
LC    AGGAGGCCTATTTAATATTATACCTGCAACCTCAACTTATTTTACGATTGGTAGTTTAGCACTAACAGGAACCCCCTTCC 13,120
WR    AGGGGGCCTATTTAATATTATACCTGCAACCTCAACTTATTTTACGATTGGTAGTTTAGCACTAACAGGAACCCCCTTCC 13,120
LWR   AGGAGGCCTATTTAATATTATACCTGCAACCTCAACTTATTTTACGATTGGTAGTTTAGCACTAACAGGAACCCCCTTCC 13,120

                 13130     13140     13150     13160     13170     13180     13190     13200
LC    TGGCAGGATTCTTCTCAAAAGACGCAATTATTGAAGCCCTAAACACCTCCCACCTAAACGCCTGAGCCCTAACCCTGACA 13,200
WR    TGGCAGGATTCTTCTCAAAAGACGCAATTATTGAAGCCCTGAACACCTCCCACCTAAACGCCTGAGCCCTAACCCTAACA 13,200
LWR   TGGCAGGATTCTTCTCAAAAGACGCAATTATTGAAGCCCTAAACACCTCCCACCTAAACGCCTGAGCCCTAACCCTGACA 13,200

                 13210     13220     13230     13240     13250     13260     13270     13280
LC    TTAATTGCCACATCATTCACCGCAGTATACAGTTTCCGATTAGTATATTTTGTAGTTATAGGAACCCCACGATTCCTGGC 13,280
WR    TTAATTGCCACATCATTCACCGCAGTATACAGTTTCCGATTAGTATATTTTGTAGTTATAGGAACCCCACGATTCCTGGC 13,280
LWR   TTAATTGCCACATCATTCACCGCAGTATACAGTTTCCGATTAGTATATTTTGTAGTTATAGGAACCCCACGATTCCTGGC 13,280

                 13290     13300     13310     13320     13330     13340     13350     13360
LC    CCTCTCCCCAATTAACGAAAATAATCCACTAGTGATTAACTCCATTAAACGACTCGCTTGAGGAAGCATTATTGCAGGCC 13,360
WR    CCTGTCCCCAATTAACGAAAATAATCCACTAGTGATTAACTCCATTAAACGACTCGCTTGAGGAAGCATTATTGCAGGCC 13,360
LWR   CCTCTCCCCAATTAACGAAAATAATCCACTAGTGATTAACTCCATTAAACGACTCGCTTGAGGAAGCATTATTGCAGGCC 13,360

                 13370     13380     13390     13400     13410     13420     13430     13440
LC    TCATTATTACACAAAACTTCCTACCAATAAAAACACCAATTATGACAATACCAGCCACCCTGAAAATAGCAGCCCTTCTC 13,440
WR    TCATTATTACACAAAACTTCCTACCAATAAAAACACCAATTATGACAATACCAACCACCCTAAAAATAGCAGCCCTTCTC 13,440
LWR   TCATTATTACACAAAACTTCCTACCAATAAAAACACCAATTATGACAATACCAGCCACCCTGAAAATAGCAGCCCTTCTC 13,440
```

```
         13450     13460     13470     13480     13490     13500     13510     13520
LC   GTAACAATTGCAGGCCTACTAGTAGCCATAGAACTAGCCAACATGACGAGCAAACAAGTAAAAGTTATTCCAATAATCCC 13,520
WR   GTAACAATTGCAGGCCTACTAGTAGCTATAGAACTAGCCAACATGACAAGCAAACAAGTAAAAATTACTCCAATAATCCC 13,520
LWR  GTAACAATTGCAGGCCTACTAGTAGCCATAGAACTAGCCAACATGACGAGCAAACAAGTAAAAGTTACTCCAATAATCCC 13,520

         13530     13540     13550     13560     13570     13580     13590     13600
LC   ATTACACCATTTCTCAAATATATTAGGATTTTTCCCCGCAATCATTCACCGACTCCTCCCAAAGCTTAAACTTACCTTAG 13,600
WR   ATTACACCATTTCTCAAATATATTAGGATTTTTCCCCGCAATCATTCACCGACTCCTCCCAAAGCTTAAACTTACCTTAG 13,600
LWR  ATTACACCATTTCTCAAATATATTAGGATTTTTCCCCGCAATCATTCACCGACTCCTCCCAAAGCTTAAACTTACCTTAG 13,600

         13610     13620     13630     13640     13650     13660     13670     13680
LC   GTCAATCAGCCGCCACTCAACTAGACAAAACGTGATTAGAAGCCATAGGACCAAAAGGCCTAGCACTAACACAAATGACC 13,680
WR   GTCAATCAGCCGCCACTCAACTAGACAAAAATGACTAGAAGCCATAGGACCAAAAGGCCTAGCACTAACACAAATGACC 13,680
LWR  GTCAATCAGCCGCCACTCAACTAGACAAAACGTGATTAGAAGCCATAGGACCAAAAGGCCTAGCACTAACACAAATGACC 13,680

         13690     13700     13710     13720     13730     13740     13750     13760
LC   ATAGCAAAAGTTACAAACGACATCTCACGAGGAATAATTAAAACATACCTAACTATTTTCCTCCTAACCCTAATTCTAGC 13,760
WR   ATAGCAAAAGTTACAAACGACATCTCACGAGGAATAATTAAAACATACCTAACTATTTTCCTCCTAACCCTAATCCTAGC 13,760
LWR  ATAGCAAAAGTTACAAACGACATCTCACGAGGAATAATTAAAACATACCTAACTATTTTCCTCCTAACCCTAATTCTAGC 13,760

         13770     13780     13790     13800     13810     13820     13830     13840
LC   CATCCTACCTGTTCTCCTCTAAACTGCACGAAGGGCCCCACGACTTAAACCACGAGTGAGTTCCAACACAACAAGCAGGG 13,840
WR   CATCCTACCTGTTCTCCTCTAAACTGCACGAAGGGCCCCACGACTTAAACCACGAGTGAGTTCCAACACAACAAGCAGGG 13,840
LWR  CATCCTACCTGTTCTCCTCTAAACTGCACGAAGGGCCCCACGACTTAAACCACGAGTGAGTTCCAACACAACAAGCAGGG 13,840

         13850     13860     13870     13880     13890     13900     13910     13920
LC   TTAAAAGCAGCACCCAAGCACAAATCACCAATATTCCCCCACCCGATGAGTATATCACAGCTACCCCACTAACATCTCCA 13,920
WR   TTAAAAGCAGCACCCAAGCACAAATCACCAATATTCCCCCACCCGATGAGTACATCACGGCCACCCCACTAACATCTCCA 13,920
LWR  TTAAAAGCAGCACCCAAGCACAAATCACCAATATTCCCCCACCCGATGAGTATATCACAGCTACCCCACTAACATCTCCA 13,920

         13930     13940     13950     13960     13970     13980     13990     14000
LC   CGTAAAACGGAGAACTCCTTCAGTCCATCAACAACCACCCACGATCCTTCATATCAGCCCCCTCAAAAGAACCCTGCCAC 14,000
WR   CGTAAAACGGAGAACTCCTTCAGTCCATCAACAACCACCCACGATCCTTCATATCAGCCCCCTCAAAAGAACCCTGCCAC 14,000
LWR  CGTAAAACAGAGAACTCCTTCAGTCCATCAACAACCACCCACGATCCTTCATATCAGCCCCCTCAAAAGAACCCTGCCAC 14,000

         14010     14020     14030     14040     14050     14060     14070     14080
LC   CAAACCAACCCCTAAAAGATATACTAACACATAACCTAAAACGGAACGACTACCCCAGGCTTCAGGATAAGGCTCGGCGG 14,080
WR   TAAACCAACCCCTAAAAGATATACTAACACGTAACCTAAAACGGAACGACTACCCCAAGCTTCAGGATAAGGCTCGGCGG 14,080
LWR  CAAACCAACCCCTAAAAGATATACTAACACATAACCTAAAACGGAACGACTACCCCAGGCTTCAGGATAAGGCTCGGCGG 14,080

         14090     14100     14110     14120     14130     14140     14150     14160
LC   CCAAAGCCGCTGAATAAGCAAAAACTACAAGCATCCCCCCTAGATAGATTAAAAAGAGAACTAAAGATAAAAAAGAGCCC 14,160
WR   CCAAAGCCGCTGAATAAGCAAAAACTACAAGCATCCCCCCTAGATAGATTAAAAAAAGAACTAAAGATAAAAAAGAGCCC 14,160
LWR  CCAAAGCCGCTGAATAAGCAAAAACTACAAGCATCCCCCCTAGATAGATTAAAAAGAGAACTAAAGATAAAAAAGAGCCC 14,160

         14170     14180     14190     14200     14210     14220     14230     14240
LC   CCATGACCAACCAAAACCCCGCATCCAACCCCAGCTGCAACCACTAAACCAAGAGCAGCAAAATAAGGAGTAGGATTAGA 14,240
WR   CCATGACCAACCAAAACCCCGCATCCAACCCCAGCTGCAACCACTAAACCAAGAGCAGCAAAATAAGGAGTAGGATTAGA 14,240
LWR  CCATGACCAACCAAAACCCCGCATCCAACCCCAGCTGCAACCACTAAACCAAGAGCAGCAAAATAAGGAGTAGGATTAGA 14,240

         14250     14260     14270     14280     14290     14300     14310     14320
LC   AGCAACAGCAACTAACCCCACAACCAAAGCTATTAATAATAAAAACATGAAATAGGTCATAATTCTTGCTCAGACTTTAA 14,320
WR   AGCAACAGCAACTAACCCCACAACCAAAGCTATTAATAATAAAAACATGAAATAGGTCATAATTCTTGCTCAGACTTTAA 14,320
LWR  AGCAACAGCAACTAACCCCACAACCAAAGCTATTAATAATAAAAACATGAAATAGGTCATAATTCTTGCTCAGACTTTAA 14,320

         14330     14340     14350     14360     14370     14380     14390     14400
LC   CCGAGACCAATGACTTGAAGAACCACCGTTGTTATTCAACTACAAGAACCATTAATGGCAAGCCTACGAAAAACACACCC 14,400
WR   CCGAGACCAATGACTTGAAGAACCACCGTTGTTATTCAACTACAAGAACCATTAATGGCAAGCCTACGAAAAACACACCC 14,400
LWR  CCGAGACCAATGACTTGAAGAACCACCGTTGTTATTCAACTACAAGAACCATTAATGGCAAGCCTACGAAAAACACACCC 14,400
```

```
        14410     14420     14430     14440     14450     14460     14470     14480
LC   CCTCATTAAAATCGCTAATGACGCACTAGTTGACCTACCCACACCATCCAACATTTCAGCATGATGAAACTTTGGCTCTC 14,480
WR   CCTCATTAAAATCGCTAATGACGCACTAGTTGACCTACCCACACCATCCAACATTTCAGCATGATGAAACTTTGGCTCTC 14,480
LWR  CCTCATTAAAATCGCTAATGACGCACTAGTTGACCTACCCACACCATCCAACATTTCAGCATGATGAAACTTTGGCTCTC 14,480

        14490     14500     14510     14520     14530     14540     14550     14560
LC   TACTAGGATTATGCTTAATTACTCAAATTCTAACCGGCCTATTTCTAGCTATACATTACACCTCAGACATTTCAACCGCA 14,560
WR   TACTAGGATTATGCTTAATTACTCAAATTCTAACCGGCCTATTTCTAGCTATACATTACACCTCAGACATTTCAACCGCA 14,560
LWR  TACTAGGATTATGCTTAATTACTCAAATTCTAACCGGCCTATTTCTAGCTATACATTACACCTCAGACATTTCAACCGCA 14,560

        14570     14580     14590     14600     14610     14620     14630     14640
LC   TTCTCATCCGTTACCCACATCTGCCGAGATGTAAACTACGGCTGACTAATTCGTAATATTCACGCCAATGGAGCATCATT 14,640
WR   TTCTCATCCGTCACCCACATCTGCCGAGATGTAAACTACGGCTGACTAATTCGTAATATTCACGCCAATGGAGCATCATT 14,640
LWR  TTCTCATCCGTTACCCACATCTGCCGAGATGTAAACTACGGCTGACTAATTCGTAATATTCACGCCAATGGAGCATCATT 14,640

        14650     14660     14670     14680     14690     14700     14710     14720
LC   CTTCTTCATCTGTATTTACATACACATTGCCCGAGGCCTATATTATGGATCATACCTTTACAAAGAAACCTGAAACATTG 14,720
WR   CTTCTTCATCTGTATTTACATACACATTGCCCGGGGCCTATATTATGGATCATACCTTTACAAAGAAACCTGAAACATTG 14,720
LWR  CTTCTTCATCTGTATTTACATACACATTGCCCGAGGCCTATATTATGGATCATACCTTTACAAAGAAACCTGAAACATTG 14,720

        14730     14740     14750     14760     14770     14780     14790     14800
LC   GAGTAGTTCTCCTACTCCTAGTTATAATGACAGCCTTTGTCGGTTATGTTCTTCCATGAGGACAAATGTCCTTTTGAGGC 14,800
WR   GAGTAGTTCTCCTACTCCTAGTTATAATGACAGCCTTTGTCGGCTATGTTCTTCCATGAGGACAAATGTCCTTTTGAGGC 14,800
LWR  GAGTAGTTCTCCTACTCCTAGTTATAATGACAGCCTTTGTCGGTTATGTTCTTCCATGAGGACAAATGTCCTTTTGAGGC 14,800

        14810     14820     14830     14840     14850     14860     14870     14880
LC   GCTACAGTAATCACAAACCTTCTATCCGCCGTGCCATATATAGGAGATATATTAGTTCAATGAATTTGAGGAGGCTTCTC 14,880
WR   GCTACAGTAATCACAAACCTTCTATCCGCCGTGCCATACATGGGAGATATATTAGTTCAATGAATTTGAGGGGGCTTCTC 14,880
LWR  GCTACAGTAATCACAAACCTTCTATCCGCCGTGCCATATATAGGAGATATATTAGTTCAATGAATTTGAGGAGGCTTCTC 14,880
```

```
        14890     14900     14910     14920     14930     14940     14950     14960
LC   CGTAGACAATGCAACATTAACACGATTCTTTGCATTTCACTTCCTTCTACCATTCATTATCGCCGCCGCTACTGTCATTC 14,960
WR   CGTAGACAATGCAACATTAACACGATTCTTCGCATTTCACTTCCTTCTACCATTCATTATCGCCGCCGCTACTGTTATTC 14,960
LWR  CGTAGACAATGCAACATTAACACGATTCTTTGCATTTCACTTCCTTCTACCATTCGTTATCGCCGCCGCTACTGTCATTC 14,960

        14970     14980     14990     15000     15010     15020     15030     15040
LC   ACCTACTGTTTCTCCACGAAACAGGATCAAATAACCCCATCGGACTGAACTCAGACGCAGACAAAATTTCTTTCCACCCA 15,040
WR   ACCTACTGTTTCTCCACGAAACAGGATCAAATAACCCCATCGGACTGAACTCAGACGCAGACAAAATTTCTTTCCACCCA 15,040
LWR  ACCTACTGTTTCTCCACGAAACAGGATCAAATAACCCCATCGGGCTGAACTCAGACGCAGACAAAATTTCTTTCCACCCA 15,040

        15050     15060     15070     15080     15090     15100     15110     15120
LC   TACTTTTCATACAAAGACCTCCTTGGGTTCGTGATTATACTACTAGCCCTCACACTCCTGGCATTATTCTCCCCAAACCT 15,120
WR   TACTTTTCATACAAAGACCTCCTTGGATTCGTGATCATACTACTAGCCCTCACACTCCTAGCATTATTCTCCCCAAACCT 15,120
LWR  TACTTTTCATACAAAGACCTCCTTGGGTTCGTGATTATACTACTAGCCCTCACACTCCTGGCATTATTCTCCCCAAACCT 15,120

        15130     15140     15150     15160     15170     15180     15190     15200
LC   TTTAGGAGACCCAGAAAACTTCACTCCAGCCAATCCCCTGGTTACTCCCCCTCATATTAAACCAGAGTGATATTTCCTGT 15,200
WR   TTTAGGAGACCCAGAAAACTTCACTCCAGCCAACCCCCTAGTTACTCCCCCTCATATTAAACCAGAATGATATTTCCTAT 15,200
LWR  TTTAGGAGACCCAGAAAACTTCACTCCAGCCAATCCCCTGGTTACTCCCCCTCATATTAAACCAGAGTGATATTTCCTAT 15,200

        15210     15220     15230     15240     15250     15260     15270     15280
LC   TTGCCTACGCCATTCTCCGATCAATTCCCAACAAACTAGGAGGAGTTCTTGCACTACTATTCTCCATCCTCGTACTAATA 15,280
WR   TTGCCTACGCCATTCTCCGATCAATTCCCAACAAGCTAGGAGGAGTTCTTGCGCTACTATTCTCTATCCTCGTACTAATA 15,280
LWR  TTGCCTACGCCATTCTCCGATCAATTCCCAACAAACTAGGAGGAGTTCTTGCACTACTATTCTCCATCCTCGTACTAATG 15,280

        15290     15300     15310     15320     15330     15340     15350     15360
LC   GTGGTACCCCTATTACATACCTCAAAACAACGAGGACTAACGTTCCGCCCAATCACCCAATTCCTATTTTGAACTCTAGT 15,360
WR   GTGGTACCCCTACTACACACCTCAAAACAACGAGGACTAACGTTCCGCCCAATCACCCAATTCCTATTTTGGACTCTAGT 15,360
LWR  GTGGTACCCCTATTACATACCTCAAAACAACGAGGACTAACGTTCCGCCCAATCACCCAATTCCTATTTTGAACTCTAGT 15,360
```

```
            15370     15380     15390     15400     15410     15420     15430     15440
LC   CGCAGACATGATTATCCTGACATGAATTGGAGGAATACCAGTAGAACATCCATTCATTATCATCGGACAAATCGCATCCG 15,440
WR   CGCGGACATGATTATCCTGACATGAATTGGAGGAATACCAGTAGAACATCCATTCATTATCATCGGACAAATCGCATCCG 15,440
LWR  CGCAGACATGATTATCCTGACATGAATTGGAGGAATACCAGTAGAACATCCATTCATTATCATCGGACAAATCGCATCCG 15,440

            15450     15460     15470     15480     15490     15500     15510     15520
LC   TCCTATATTTCGCACTGTTCCTTGTTCTCTTCCCACTAGCAGGATGATTAGAAAATAAAGCACTGAAATGAGCTTGCCCT 15,520
WR   TCCTATATTTCGCACTATTCCTTGTTCTCTTCCCACTAGCAGGATGATTAGAAAATAAAGCACTGAAATGAGCTTGCCCT 15,520
LWR  TCCTATATTTCGCACTGTTCCTTGTTCTCTTCCCACTAGCAGGATGATTAGAAAATAAAGCACTGAAATGAGCTTGCCCT 15,520

            15530     15540     15550     15560     15570     15580     15590     15600
LC   AGTAGCTTAGCATAAAAGCATCGGTCTTGTAATCCGAAGATCGGAGGTTAAATTCCTCCCTAGCGCCCAGAAAAGAGAGA 15,600
WR   AGTAGCTTAGTATAAAAGCATCGGTCTTGTAATCCGAAGATCGGAGGTTAAATTCCTCCCTAGCGCCCAGAAAAGAGAGA 15,600
LWR  AGTAGCTTAGCATAAAAGCATCGGTCTTGTAATCCGAAGATCGGAGGTTAAATTCCTCCCTAGCGCCCAGAAAAGAGAGA 15,600

            15610     15620     15630     15640     15650     15660     15670     15680
LC   TTTTAACTCTCACCCCTGGCTCCCAAAGCCAGAATTCTAAACTAAACTATTTTCTGGGGATAACCATCCCTGTATGGTTT 15,680
WR   TTTTAACTCTCACCCCTGGCTCCCAAAGCCAGAATTCTAAACTAAACTATTTTCTGGGGATAACCATCCCTGTATGGTTT 15,680
LWR  TTTTAACTCTCACCCCTGGCTCCCAAAGCCAGAATTCTAAACTAAACTATTTTCTGGGGATAACCATCCCTGTATGGTTT 15,680

            15690     15700     15710     15720     15730     15740     15750     15760
LC   AATGCATAATATGCATAATATTACATTAGTGTATTAGTACATATATGTATTATCACCATATCATTATATTAACCCCAAAG 15,760
WR   AATACATAATATGCATAATATTACATTAGTGTATTAGTACATATATGTATTATCACCATATCATTATTTTAACCCCAAAG 15,760
LWR  AATACATAATATGCATAATATTACATTAGTGTATTAGTACATATATGTATTATCACCATATCATTATATTAACCCCAAAG 15,760

            15770     15780     15790     15800     15810     15820     15830     15840
LC   CAAGTACATATAAACTAAGGTATGCATAAAGCATAATCTTAAGACTCACAAGTTAAATTATTTTAACCCGGGTAATATAT 15,840
WR   CAAGTACATATAAACTAAGGTATGCATAAAGCATAATCTTAAGACTCACAAGTTAAATTATTTTAACCCGGGTAATATAT 15,840
LWR  CAAGTACATATAAACTAAGGTATGCATAAAGCATAATCTTAAGACTCACAAGTTAAATTATTTTAACCCGGGTAATATAT 15,840

            15850     15860     15870     15880     15890     15900     15910     15920
LC   TATTCCCCAAGAAATTGTCCCCACATTTTTCCTTGAATGACTCAACTAAGGTTTTATTCAAACATATTAATGTAGTAAGA 15,920
WR   TATTCCCCAAGAAATTGTCCTCACATTTTTCCTTGAATGATTCAGCTAAGGTTTTATTCGAACATATTAATGTAGTAAGA 15,920
LWR  TATTCCCCAAGAAATTGTCCCCACATTTTTCCTTGAATGACTCAACTAAGGTTTTATTCAAACATATTAATGTAGTAAGA 15,920

            15930     15940     15950     15960     15970     15980     15990     16000
LC   GACCACCAACC-ATTTATATAAAGGAATATCATGCATGATAGAATCAGGGACATCAATTGTGGGGGTCGCACAATATGAA 15,999
WR   AACCACCAACTAATTTACATAAAGGAATATCATGCATGATGGAATCAGGGACACCAACTGTGGGGGTTGCACAATGTGAA 16,000
LWR  GACCACCAACC-ATTTATATAAAGGAATATCATGCATGATAGAATCAGGGACATCAATTGTGGGGGTCGCACAATATGAA 15,999

            16010     16020     16030     16040     16050     16060     16070     16080
LC   CTATTACTGGCATCTGGTTCCTATTTCAGGTACATAACTGTAATACTCCACCCTCGGATAATTATACTGGCATCTGATTA 16,079
WR   CTATTACTGGCATCTGGTTCCTATTTCAGGTACATAACTGTAATACTCCACCCTCGGATAATTATACTGGCATCTGATTA 16,080
LWR  CTATTACTGGCATCTGGTTCCTATTTCAGGTACATAACTGTAATACTCCACCCTCGGATAATTATACTGGCATCTGATTA 16,079

            16090     16100     16110     16120     16130     16140     16150     16160
LC   ATGGTGTGGTACATATGGTTCATTACCCCACATGCCGAGCATTCTTTTATATGCATAAGGTATTTTTTTTTTGGTTTCCT 16,159
WR   ATGGTGTAGTACATATGGTTCATTACCCCACATGCCGAGCATTCTTTTATATGCATAAGGTATTTTTTTTTTGGTTTCCT 16,160
LWR  ATGGTGTGGTACATATGGTTCATTACCCCACATGCCGAGCATTCTTTTATATGCATAAGGTATTTTTTTTTTGGTTTCCT 16,159

            16170     16180     16190     16200     16210     16220     16230     16240
LC   TTCATCTTGCATCTCAGAGTGCAGGCACAAATGTTGGTTTAAGGTTGAACATTTTCCTTGAATGTGATTATATAAATGAA 16,239
WR   TTCATCTTGCATCTCAGAGTGCAGGCACAAATGTTGGTTTAAGGTTGAACATTTTCCTTGAATGTGATTATATAAATGAA 16,240
LWR  TTCATCTTGCATCTCAGAGTGCAGGCACAAATGTTGGTTTAAGGTTGAACATTTTCCTTGAATGTGATTATATAAATGAA 16,239

            16250     16260     16270     16280     16290     16300     16310     16320
LC   TTATCGTAAGACATAATTTAAGAACTGCATACTTCTAACTCAAGTGCATAACATATTCATCTCTTATTCAACTTATCCTT 16,319
WR   TTATCGTAAGACATAATTTAAGAACTGCATACTTCTAACTCAAGTGCATAACATATTCATCTCTTATTCAACTTATCCTT 16,320
LWR  TTATCGTAAGACATAATTTAAGAACTGCATACTTCTAACTCAAGTGCATAACATATTCATCTCTTATTCAACTTATCCTT 16,319

            16330     16340     16350     16360     16370     16380     16390     16400
LC   ATATAGTGCCCCCTTTGGTTTTTGCGCGACAAACCCCCCTACCCCCTACGCTCAAAGAATCCTGTTATCCTTGTCAAACC 16,399
WR   ATATAGTGCCCCCTTTGGTTTTTGCGCGACAAACCCCCCTACCCCCTACGCTCAAAGAATCCTGTTATCCTTGTCAAACC 16,400
LWR  ATATAGTGCCCCCTTTGGTTTTTGCGCGACAAACCCCCCTACCCCCTACGCTCAAAGAATCCTGTTATCCTTGTCAAACC 16,399

            16410     16420     16430     16440     16450     16460     16470     16480
LC   CCGAAACCAAGGAGGACCCAAGAACGTGTAAGCCAACGAGTTGAGGTACGAATTGGCATCCCATTATATATATATATATA 16,479
WR   CCGAAACCAAGGAGGACCCAAGAACGTGTAAGCCAACGAGTTGAGGTACGAATTGGCATCCCATTATATATATATATATA 16,480
LWR  CCGAAACCAAGGAGGACCCAAGAACGTGTAAGCCAACGAGTTGAGGTACGAATTGGCATCCCATTATATATATATATATA 16,479

            16490     16500     16510     16520     16530     16540     16550     16560
LC   TATGTGCATCGGTTTTTTTATCCCAATTCAGTAATCACCCAAAAATCTCTGCCAAAAACCCCAAAAAATCACCTCCACAC 16,559
WR   TATGTGCATCGGTTTTTTTATCACAATTCAGTGATCACCTAAAAATCTCTGCCAAAAACCCCAAAAAATCACCTCCACAC 16,560
LWR  TATGTGCATCGGTTTTTTTATCCCAATTCAGTAATCACCCAAAAATCTCTGCCAAAAACCCCAAAAAATCACCTCCACAC 16,559

            16570     16580
LC   TAAATTTTCTAACATTATTTA 16,580
WR   TAAATTTTCTAACA-TATTTA 16,580
LWR  TAAATTTTCTAACATTATTTA 16,580
```

Figure S2. Complete mtDNA sequences of LC, WR, and LWR.
